# Platelet P2Y_1_ receptor exhibits constitutive G protein signaling and β-arrestin 2 recruitment

**DOI:** 10.1186/s12915-023-01528-y

**Published:** 2023-02-01

**Authors:** Agnès Ribes, Cédric Garcia, Marie-Pierre Gratacap, Evi Kostenis, Laurent O. Martinez, Bernard Payrastre, Jean-Michel Sénard, Céline Galés, Véronique Pons

**Affiliations:** 1 Laboratoire d’Hématologie, Centre Hospitalier Universitaire de Toulouse, F-31000 Toulouse, France; 2 INSERM, UMR 1297, Institut des Maladies Métaboliques et Cardiovasculaires, Université de Toulouse, F-31432 Toulouse, France; 3 Molecular, Cellular and Pharmacobiology Section, Institute for Pharmaceutical Biology, University of Bonn, Nussallee 6, 53115 Bonn, Germany; 4 Service de Pharmacologie Clinique, Centre Hospitalier Universitaire de Toulouse, Faculté de Médecine, Université de Toulouse, F-31000 Toulouse, France

**Keywords:** GPCR, Constitutive signaling, Inverse agonism, MRS2179, P2Y receptor

## Abstract

**Background:**

Purinergic P2Y_1_ and P2Y_12_ receptors (P2Y_1_-R and P2Y_12_-R) are G protein-coupled receptors (GPCR) activated by adenosine diphosphate (ADP) to mediate platelet activation, thereby playing a pivotal role in hemostasis and thrombosis. While P2Y_12_-R is the major target of antiplatelet drugs, no P2Y_1_-R antagonist has yet been developed for clinical use. However, accumulating data suggest that P2Y_1_-R inhibition would ensure efficient platelet inhibition with minimal effects on bleeding. In this context, an accurate characterization of P2Y_1_-R antagonists constitutes an important preliminary step.

**Results:**

Here, we investigated the pharmacology of P2Y_1_-R signaling through Gq and β-arrestin pathways in HEK293T cells and in mouse and human platelets using highly sensitive resonance energy transfer-based technologies (BRET/HTRF). We demonstrated that at basal state, in the absence of agonist ligand, P2Y_1_-R activates Gq protein signaling in HEK293T cells and in mouse and human platelets, indicating that P2Y_1_-R is constitutively active in physiological conditions. We showed that P2Y_1_-R also promotes constitutive recruitment of β-arrestin 2 in HEK293T cells. Moreover, the P2Y_1_-R antagonists MRS2179, MRS2279 and MRS2500 abolished the receptor dependent-constitutive activation, thus behaving as inverse agonists.

**Conclusions:**

This study sheds new light on P2Y_1_-R pharmacology, highlighting for the first time the existence of a constitutively active P2Y_1_-R population in human platelets. Given the recent interest of P2Y_12_-R constitutive activity in patients with diabetes, this study suggests that modification of constitutive P2Y_1_-R signaling might be involved in pathological conditions, including bleeding syndrome or high susceptibility to thrombotic risk. Thus, targeting platelet P2Y_1_-R constitutive activation might be a promising and powerful strategy for future antiplatelet therapy.

**Supplementary Information:**

The online version contains supplementary material available at 10.1186/s12915-023-01528-y.

## Background

Co-activation of both P2Y_1_-R and P2Y_12_-R is necessary for full platelet aggregation by ADP [1]. P2Y_1_-R is responsible for platelet shape change and initiates small and reversible ADP-induced platelet aggregation by triggering Gq-dependent phospholipase C (PLC) activation leading to inositol triphosphate (IP3) production and subsequent calcium release into the cytoplasm [2]. P2Y_12_-R activation results in amplification and stabilization of the aggregation response, through Gi-dependent adenylyl cyclase inhibition and subsequent cyclic adenosine monophosphate (cAMP) decrease [3]. Antiplatelet drugs that target P2Y_12_-R activation have been extensively developed and are widely used in the treatment and prevention of arterial thrombosis [4, 5], but, by contrast, no selective P2Y_1_-R antagonist have yet been developed for clinical use. One reason for this is that while P2Y_12_-R expression is mostly restricted to platelets, P2Y_1_-R exhibits a broader expression pattern, thereby raising concerns about P2Y_1_-R inhibition and possible unforeseen outcomes. However, P2Y_1_-R-null mice are viable with no apparent abnormalities, thus suggesting that P2Y_1_-R might be a promising target for the development of antiplatelet drugs. Indeed, platelets from P2Y_1_-R-deficient mice exhibited impaired platelet aggregation in response to ADP and a strong resistance to thrombosis [6, 7]. Moreover, selective inhibition of P2Y_1_-R in rats using P2Y_1_-R antagonists reduced both venous and arterial thrombosis [8]. Importantly, P2Y_1_-R inhibition resulted in only moderate prolongation of the bleeding time [9, 10], making it a good candidate for inhibiting platelet activation with presumably less bleeding outcome while bleeding risk is the major drawback of anti-P2Y_12_-R therapies. Indeed, P2Y_12_-R blockers face some limitations as irreversible platelet inhibition achieved by thienopyridines (clopidogrel, prasugrel) displayed a delayed onset of action and increased the risk of bleeding [11], while reversible binding drugs such as ticagrelor and cangrelor have been recently associated with adverse effects [12, 13].

Recently, the diadenosine tetraphosphate derivative GLS-409 designed to achieve dual inhibition of both P2Y_1_-R and P2Y_12_-R was shown to promote potent inhibition of canine coronary artery thrombosis and reversible human platelet inhibition [14, 15]. Thus, developing selective P2Y_1_-R-targeting drugs might be a novel and promising antithrombotic strategy to ensure efficient inhibition of platelet aggregation with a minimal effect on bleeding.

Interestingly, we and others recently demonstrated that P2Y_12_-R exhibited constitutive activity on Gi/o proteins and downstream adenylyl cyclase inhibition in human resting platelets [16, 17]. At resting state, this constitutive P2Y_12_-R signaling might be essential for platelets to respond rapidly to a vessel injury, by lowering cAMP levels and sensitizing platelets prior to activation. Importantly, P2Y_12_-R constitutive signaling needs to be fine-tuned to ensure proper control of hemostasis since loss of P2Y_12_-R constitutive activity was associated with bleeding syndrome [17] while enhanced P2Y_12_-R constitutive signaling was correlated with platelet hyperactivity in diabetes [18]. Similarly, transgenic mice expressing constitutively active P2Y_12_-R chimera exhibited increased platelet activation and thrombosis [19], highlighting the pro-thrombotic role of the P2Y_12_-R constitutive activity. Therefore, monitoring platelet P2Y_12_-R constitutive activity might be a powerful readout to evaluate the thrombotic status of patients and thus adjust the antiplatelet therapy by balancing the antithrombotic beneficial effects with the bleeding risk. In this context, the identification of inverse agonists might be a promising avenue for the development of new therapeutic molecules able to precisely modulate the constitutive activity of P2Y_12_-R and ultimately prevent thrombosis. Interestingly, many P2Y_12_-R antagonists (AR-C78511, cangrelor, ticagrelor, selatogrel) were indeed described as inverse agonists at P2Y_12_-R constitutive signaling [16–20], suggesting that the clinical benefits of antiplatelet drugs might be directly related to inverse agonism at P2Y_12_-R.

Since P2Y_1_-R is a good candidate for inhibiting platelet activation with presumably less bleeding outcome, here we characterized the pharmacological properties of P2Y_1_-R regarding the receptor constitutive activity. We demonstrated that much like P2Y_12_-R on Gi/o protein signaling, P2Y_1_-R exhibited constitutive activity leading to Gq protein activation and downstream PLC/IP3 signaling both in HEK293T cells and in mouse and human resting platelets. In addition to G protein-dependent signaling, we also investigated β-arrestin 2 recruitment as β-arrestin 2 plays a prominent role in platelet GPCR signaling. In marked contrast with P2Y_12_-R, we showed that P2Y_1_-R also displayed constitutive association with β-arrestin 2 in HEK293T cells, highlighting a constitutive recruitment of β-arrestin 2. Interestingly, the P2Y_1_-R antagonists, MRS2179, MRS2279, and MRS2500, acted as an inverse agonist at P2Y_1_-R by counteracting both constitutive G protein signaling and β-arrestin 2 recruitment to the receptor.

Altogether, our data provide new critical insights toward P2Y_1_-R pharmacological characterization, highlighting for the first time the constitutive activity of this receptor and inverse agonism in resting human platelets. Importantly, the level of agonist-independent P2Y_1_-R and P2Y_12_-R basal signaling might be directly correlated with the platelet responsiveness and therefore represent a promising readout to evaluate the thrombotic risk.

## Results

### P2Y_1_-R exhibits constitutive G𝛼q-dependent signaling in HEK293T cells

**Fig. 1:**
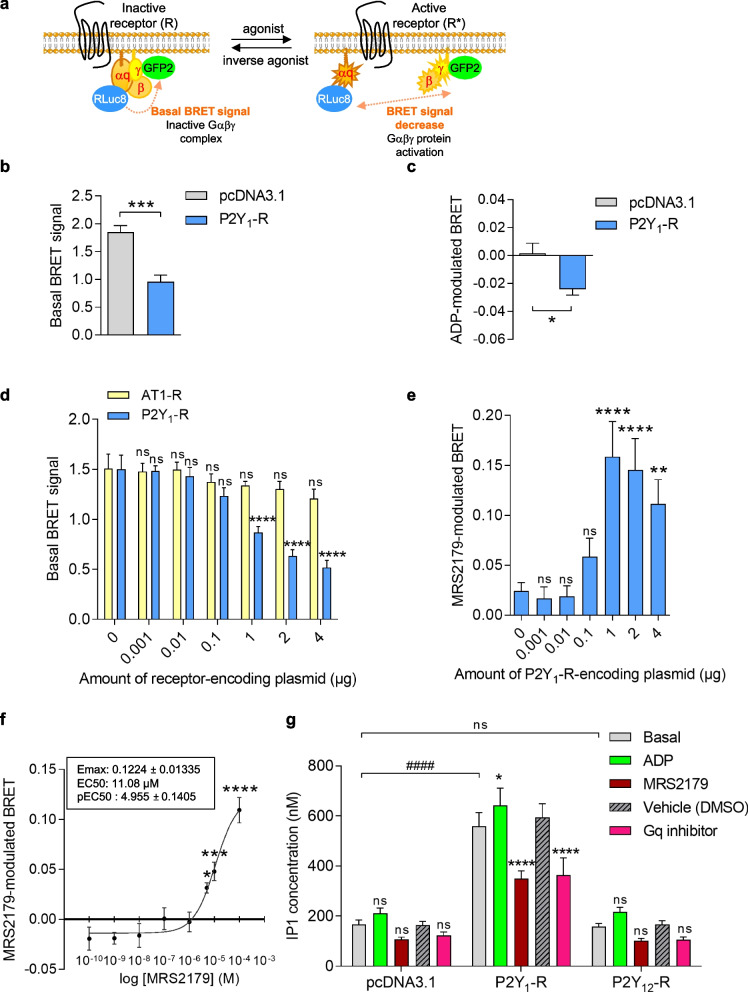
P2Y_1_-R constitutively activates Gq protein-dependent signaling in HEK293T cells. **a** Schematic representation depicting the BRET signal measured at basal state, reflecting the inactive Gαqβ1γ2 complex, and resulting from an energy transfer between the energy donor RLuc8 fused to Gαq protein and the energy acceptor GFP2 fused to Gγ2 protein. Agonist-induced or constitutive receptor activation will promote G protein activation and dissociation that is reflected by a decrease of the BRET signal. **b** Basal Gαq protein activation was evaluated by measuring basal BRET signal in HEK293T cells co-expressing Gαq-RLuc8, GFP2-Gγ2, and Gβ1 in the absence (pcDNA3.1) or in the presence of P2Y_1_-R. Data represent the mean ± s.e.m. of six independent experiments and statistical significance between cells expressing P2Y_1_-R or not was assessed using an unpaired t-test (***p < 0.001). **c** Gαq protein activation was evaluated by measuring BRET signal in HEK293T cells co-expressing Gαq-RLuc8, GFP2-Gγ2, and Gβ1 in the absence (pcDNA3.1) or in the presence of P2Y_1_-R, after stimulation or not with ADP (10 μM) for 1 min. Results are expressed as the difference in the BRET signal measured in the presence and in the absence of ADP. Data represent the mean ± s.e.m. of six independent experiments and statistical significance of ADP-induced BRET modulation between cells expressing P2Y_1_-R or not was assessed using an unpaired *t*-test (*p < 0.05). **d** Basal Gαq protein activation was evaluated by measuring basal BRET signal in HEK293T cells co-expressing Gαq-RLuc8, GFP2-Gγ2, and Gβ1 in the absence (0 μg) or in the presence of increasing amounts of vectors (ranging from 0.001 to 4 μg/dish) encoding AT1-R or P2Y_1_-R. Data represent the mean ± s.e.m. of five independent experiments and statistical significance between cells expressing receptors or not (0 μg) was assessed using two-way ANOVA followed by Sidak’s post-tests (****p < 0.0001; ns, not statistically significant). **e** Gαq protein activation was evaluated by measuring BRET signal in HEK293T cells co-expressing Gαq-RLuc8, GFP2-Gγ2, and Gβ1 in the absence (0 μg) or in the presence of increasing amounts of vectors encoding P2Y_1_-R (ranging from 0.001 to 4 μg/dish), after stimulation or not with MRS2179 (10 μM) for 1 min. Results are expressed as the difference in the BRET signal measured in the presence and in the absence of MRS2179. Data represent the mean ± s.e.m. of seven independent experiments and statistical significance of MRS2179-induced BRET modulation between cells expressing P2Y_1_-R or not (0 μg) was assessed using one-way ANOVA followed by Holm-Sidak’s post-tests. (**p < 0.01; ****p < 0.0001; ns, not statistically significant). **f** Dose-response curve was performed in HEK293T cells co-expressing Gαq-RLuc8, GFP2-Gγ2, Gβ1, and P2Y_1_-R after stimulation or not with increasing concentrations of MRS2179 for 1 min. Results are expressed as the difference in the BRET signal measured in the presence and in the absence of MRS2179. Data represent the mean ± s.e.m. of five independent experiments. Statistical significance between unstimulated and stimulated cells was assessed by one-way ANOVA followed by Sidak’s post-tests (*p < 0.05; ***p < 0.001; ****p < 0.0001). Maximal efficacy (Emax ± s.e.m.) and potency (EC50 and pEC50 ± s.e.m.) of MRS2179 are indicated in the inset. **g** HEK293T cells expressing P2Y_1_-R, P2Y_12_-R, or not (pcDNA3.1) were incubated in the absence (basal) or in the presence of ADP (100 μM), MRS2179 (10 μM), vehicle (DMSO) ,or the Gq inhibitor (100 nM) for 2 h and IP1 accumulation was quantified. Data represent the mean ± s.e.m. of five independent experiments and are expressed as IP1 concentration (nM). The statistical comparison between unstimulated (basal or vehicule (DMSO)) and stimulated (ADP, MRS2179, or Gq inhibitor) cells or between cells expressing the different receptors was assessed using two-way ANOVA followed by Bonferroni’s or Dunnett’s post-tests respectively (*p < 0.05; **** or ^####^*p* < 0.0001; ns, not statistically significant)

Since P2Y_12_-R was recently shown to exhibit constitutive activity on Gαi/o protein signaling in human resting platelets [16–18], we investigated whether P2Y_1_-R would display ligand-independent activation as well. As previously described [17, 21, 22], using a BRET^2^ (bioluminescence resonance energy transfer)-based assay, we directly monitored the basal Gαq protein activation by measuring the interaction between Gαq and Gγ2 subunits of the Gαqβ1γ2 heterotrimer complex. Indeed, this assay is based on the non-radiative transfer between the Renilla reniformis luciferase (RLuc8) energy donor fused to the Gαq protein subunit and the fluorescent GFP2 energy acceptor fused to Gγ2 protein subunit (Fig. 1a). At basal state, in the absence of agonist, the preassembled inactive Gαqβ1γ2 heterotrimeric complex will favor the detection of a basal BRET signal due to the close proximity between Gαq and Gγ2 protein subunits. By contrast, ligand-independent constitutive receptor activation or agonist-induced receptor activation will promote Gαq/Gγ2 subunit dissociation, thereby leading to a decrease of the basal BRET signal (Fig. 1a). At basal state, a high basal BRET signal was detected in control cells (pcDNA3.1), indicative of Gαq/Gγ2 proximity and Gαqβ1γ2 inactive complex (Fig. 1b). By contrast, at comparable Gαq protein BRET probe expression (Additional File 1: Fig. S1a), expression of P2Y_1_-R promoted a strong and significant decrease of the basal BRET signal, suggesting that P2Y_1_-R constitutively activated Gαq protein (Fig. 1b). To preclude any P2Y_1_-R activation by a passive release of ADP by HEK293T cells, we performed similar experiments in the presence of high concentration of apyrase (0.2U/mL) to degrade any trace of ADP in the medium (Additional File 2: Fig. S2a). Under these conditions, P2Y_1_-R expression still induces a marked and significant decrease of the basal BRET signal compared to control cells (pcDNA3.1), thereby supporting a P2Y_1_-R-dependent constitutive activation of Gαq protein. Surprisingly, ADP only triggered a significant but moderate decrease of the BRET signal (Fig. 1c), indicating a weak Gαq protein activation following P2Y_1_-R stimulation. These results suggested that, in these experimental conditions, P2Y_1_-R exhibited a strong constitutive activity on Gαq protein signaling, thereby narrowing ADP-dependent receptor activation, as previously described for P2Y_12_-R on Gαi/o protein signaling [17]. Interestingly, the constitutive activity is a specific feature of P2Y_1_-R since the basal BRET signal was gradually decreased with increasing cell surface expression of P2Y_1_-R (Additional File 3: Fig. S3a, left panel) while, at similar Gαq protein BRET probe expression levels (Additional File 1: Fig. S1b), it was not significantly impacted after increasing plasma membrane expression of another Gαq protein-coupled receptor, the angiotensin II type 1 receptor (AT1-R) (Fig. 1d and Additional File 3: Fig. S3a, right panel). Of note, because P2Y_1_-R and AT1-R were respectively Myc- and HA-tagged and were thus detected using different primary antibodies, their expression at the cell surface cannot be compared between each other’s. These results demonstrate that, contrary to AT1-R, P2Y_1_-R displays constitutive activation of Gαq protein in HEK293T cells and that this ligand-independent basal activation correlates with the receptor expression level, in agreement with the well-known receptor expression dependency of GPCR constitutive activity [23].

Since P2Y_12_-R antagonists such as ticagrelor and cangrelor were shown to target the receptor constitutive activity, thereby acting as inverse agonists at P2Y_12_-R [16, 17], we then investigated the pharmacological properties of a P2Y_1_-R antagonist, MRS2179 [24], regarding the constitutive activity of P2Y_1_-R on Gαq protein activation. MRS2179 stimulation induced an increase of the BRET signal of G protein biosensors in cells expressing P2Y_1_-R (Fig. 1e), indicating that MRS2179 counteracted the constitutive activation of P2Y_1_-R, thus behaving as an inverse agonist. Indeed, inverse agonists inhibit the constitutive activity of a receptor, by switching the receptor from an active R* to an inactive R conformational state. Consequently, inverse agonists should be prone to increase the BRET signal between Gαq and Gγ2 BRET biosensors, reflecting Gαq/Gγ2 reassembly (Fig. 1a) [17, 25]. This inhibition on P2Y_1_-R constitutive activity was dependent on receptor amount since MRS2179-modulated BRET signal increased with cell surface P2Y_1_-R expression level (Fig. 1e and Additional File 3: Fig. S3a, left panel), while Gαq protein BRET probe expression remained constant (Additional File 1: Fig. S1c). Noticeably, MRS2179 exhibited significant inverse agonist efficacy at the highest P2Y_1_-R expression levels (Fig. 1e), in agreement with the Gαq protein constitutive activation detected at similar receptor expression levels (Fig. 1d). To gain more insight into the inverse agonist efficacy of MRS2179 at P2Y_1_-R, we performed a concentration-response curve on Gαq protein activity and observed that MRS2179 exhibited a relatively low potency (EC50 = 11.08 μM) (Fig. 1f).

We then further explored the P2Y_1_-R constitutive activity by monitoring downstream Gαq-dependent signaling in P2Y_1_-R-expressing HEK293T cells (Fig. 1g). Since Gαq protein activation is known to induce PLC activation leading to IP3 and subsequent cytosolic calcium release, we analyzed the constitutive Gαq-dependent P2Y_1_-R signaling by quantifying intracellular inositol monophosphate (IP1) using a monoclonal antibody-based competitive ELISA. IP1 is a downstream metabolite of IP3 that accumulates in cells following Gαq protein-coupled receptor activation, making it an ideal readout for Gαq protein-dependent signaling pathways. Interestingly, at basal state, while more expressed than P2Y_1_-R at the plasma membrane (Additional File 3: Fig. S3b), P2Y_12_-R did not impact IP1 levels compared to control cells (pcDNA3.1) while P2Y_1_-R markedly increased IP1 production (Fig. 1g), suggesting a specific constitutive activity of P2Y_1_-R on PLC/IP3 pathway, in agreement with the constitutive activity detected on the Gαq protein (Fig. 1b). Once again, this basal P2Y_1_-R-dependent IP1 production cannot be due to the presence of ADP in the medium since it was still detected in P2Y_1_-R expressing cells in the presence of high apyrase concentration (0.2U/mL), even after 30-min accumulation (Additional File 2: Fig. S2b). Interestingly, IP1 production was slightly but significantly potentiated upon ADP stimulation in cells expressing P2Y_1_-R, suggesting that under these conditions, a part of P2Y_1_-R was still in an inactive R conformation that is sensitive to ADP stimulation, in agreement with the detection of ADP-promoted Gαq protein activation (Fig. 1c). P2Y_1_-R-mediated constitutive IP1 production was strongly inhibited following MRS2179 treatment, thus demonstrating that MRS2179 behaved as an inverse agonist on constitutive Gq/PLC/IP3 signaling (Fig. 1g). Consistently, the plant-derived Gαq inhibitor (FR900359) [26] also significantly reduced the constitutive P2Y_1_-R-dependent IP1 production (Fig. 1g).

Altogether, these results strongly support that P2Y_1_-R constitutively activates the Gq/PLC-dependent signaling in HEK293T cells and that MRS2179 acts as an inverse agonist on this P2Y_1_-R constitutive signaling (Fig. 1).

### Platelet P2Y_1_-R constitutive signaling is counteracted by MRS2179 inverse agonist

**Fig. 2:**
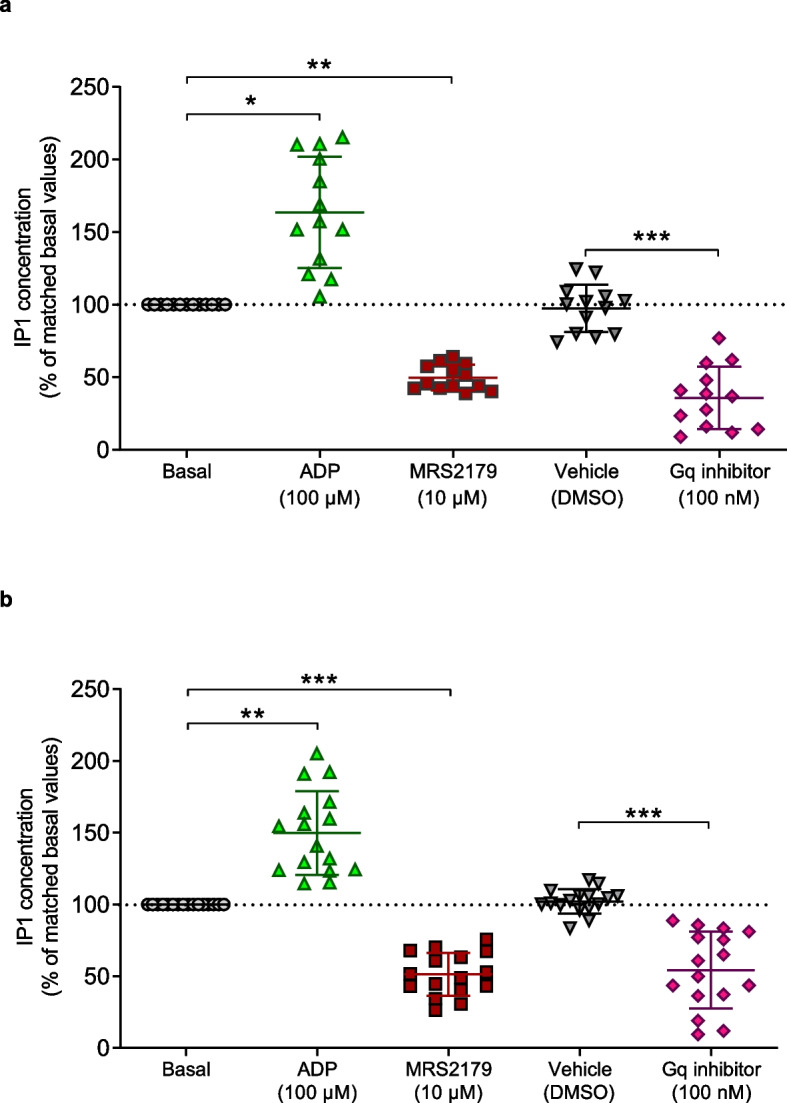
P2Y_1_-R exhibits constitutive signaling in resting mouse and human platelets. **a**, **b** Washed murine (**a**) or human (**b**) platelets were incubated in the absence (basal, dashed line) or in the presence of ADP (100 μM), MRS2179 (10 μM), vehicle (DMSO), or the Gq inhibitor (100 nM) for 2 h and IP1 accumulation was quantified. Data represent the mean ± s.d. of 13 mice (**a**) or 16 healthy donors (**b**) and are expressed as the percentage of matched basal values. The statistical comparison between untreated (basal or vehicle) and treated (ADP, MRS2179 or Gq inhibitor respectively) platelets was assessed using Kruskal-Wallis test followed by Dunn’s post-tests (*p < 0.05; **p < 0.01; ***p < 0.001)

Since our data demonstrated that P2Y_1_-R exhibits constitutive ADP-independent signaling in HEK3293T cells (Fig. 1), we investigated the relevance of such P2Y_1_-R constitutive activity and inverse agonist efficacy of MRS2179 in both murine (Fig. 2a) and human (Fig. 2b) washed platelets, in the presence of apyrase (0.02 U/mL) to degrade any trace of ADP and indomethacin to prevent thromboxane A2 production. As performed in HEK293T cells, we thus quantified intracellular IP1 levels to monitor Gαq protein-dependent signaling pathways. We observed that MRS2179 decreased IP1 levels, thus behaving as an inverse agonist and thereby demonstrating a basal IP1 production in the absence of agonist. These results indicate the existence of a P2Y_1_-R constitutive activation of Gαq signaling in murine (Fig. 2a) and human (Fig. 2b) resting platelets. P2Y_1_-R constitutive activity was still detected in human platelets incubated with higher apyrase concentration (0.2 U/mL), indicating that the basal IP1 production did not result from platelet activation and ADP release (Additional File 4: Fig. S4a). Accordingly, we demonstrated that dense granule exocytosis and ADP release did not occur in washed human platelets since when assessing surface expression of platelet CD63 activation marker in resting and activated platelets, we showed that stimulation with thrombin receptor activating peptide (TRAP) was able to trigger a strong exposure of CD63 at the platelet surface (Additional File 4: Fig. S4b). Consistently with what we observed in P2Y_1_-R expressing HEK293T cells (Fig. 1g), the basal IP1 production was also inhibited by the Gαq inhibitor in both mouse (Fig. 2a) and human (Fig. 2b) washed platelets. Notably, IP1 production was significantly increased upon ADP stimulation in murine and human platelets, much like in P2Y_1_-R expressing HEK293T cells, suggesting the existence of two P2Y_1_-R populations at resting state: one “pre-active” ADP-insensitive receptor population exhibiting constitutive activity and another “inactive” receptor population that is responsive to ADP.

Altogether, these data highlighted the constitutive activity at P2Y_1_-R/Gαq pathways in mouse and human resting platelets and demonstrated that MRS2179 displayed inverse agonist efficacy by counteracting P2Y_1_-R-dependent signaling.

### P2Y_1_-R is constitutively associated with β-arrestin 2

In addition to G protein-dependent signaling, GPCRs elicit β-arrestin-dependent signaling pathways. Since β-arrestin 2 was involved in platelet GPCR desensitization, we then investigated β-arrestin 2 recruitment following P2Y_1_-R activation.

**Fig. 3:**
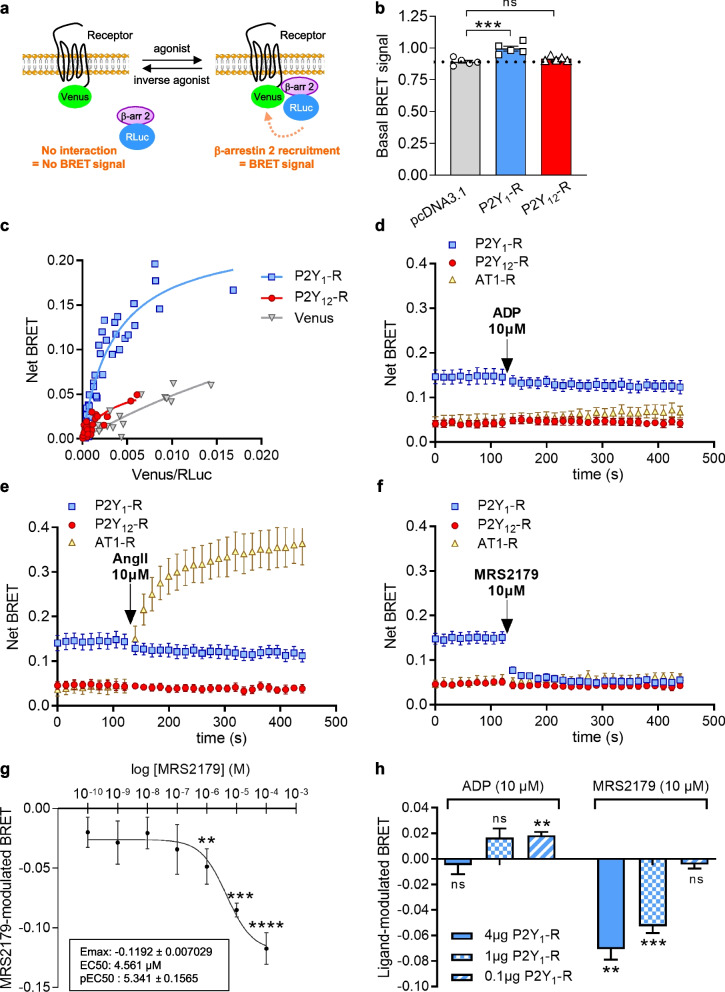
P2Y_1_-R triggers constitutive recruitment of β-arrestin 2. **a** Schematic representation depicting the BRET signal measured between β-arrestin 2 fused to the energy donor RLuc and the receptor fused to the energy acceptor Venus. The recruitment of β-arrestin 2 to the receptor in the presence of an agonist will promote an increase of the BRET signal compared to the basal state as the proximity between the β-arrestin 2 energy donor and the receptor energy acceptor increased. **b** Basal BRET signal was evaluated in HEK293T cells expressing β-arrestin 2-RLuc alone (pcDNA3.1) or in the presence of P2Y_1_-R-Venus, or P2Y_12_-R-Venus. Data represent the mean ± s.e.m. of five independent experiments and statistical significance between cells expressing receptors or not was assessed using one-way ANOVA followed by Dunnett’s post-tests (***p < 0.001; ns, not statistically significant). **c** Basal BRET signal was measured in HEK293T cells co-expressing a fixed amount of β-arrestin 2-RLuc and increasing amounts of P2Y_1_-R-Venus, P2Y_12_-R-Venus, or Venus. Results are expressed as the Net BRET and were analyzed by nonlinear regression on a pooled data set from five independent experiments assuming a model with one-site binding. **d**–**f** β-arrestin 2 recruitment was evaluated by monitoring BRET signal in HEK293T cells co-expressing β-arrestin 2-RLuc and P2Y_1_-R-Venus, P2Y_12_-R-Venus, or AT1-R-Venus, after stimulation or not with ADP (10 μM) (**d**), AngII (10 μM) (**e**), or MRS2179 (10 μM) (**f**) for 5 min. The 2 first minutes represent the BRET signal at basal state, before injecting the ligand. Results are expressed as the Net BRET and data represent the mean ± s.e.m. of five independent experiments. **g** Dose-response curve was performed in HEK293T cells co-expressing β-arrestin 2-RLuc and P2Y_1_-R-Venus after stimulation or not with increasing concentrations of MRS2179 for 15 min. Results are expressed as the difference in the BRET signal measured in the presence and in the absence of MRS2179. Data represent the mean ± s.d. of six independent experiments. Statistical significance between unstimulated and stimulated cells was assessed by Friedman test followed by Dunn’s post-tests (**p < 0.01; ***p < 0.001; ****p < 0.0001). Maximal efficacy (Emax) and potency (EC50 and pEC50 ± s.e.m.) of MRS2179 are indicated in the inset. **h** β-arrestin 2 recruitment was evaluated by monitoring BRET signal in HEK293T cells co-expressing β-arrestin 2-RLuc and decreasing amounts of vectors encoding P2Y_1_-R-Venus after stimulation or not with ADP (10 μM) or MRS2179 (10 μM) for 15 min. Results are expressed as the difference in the BRET signal measured in the presence and in the absence of ligand. Data represent the mean ± s.e.m. of five independent experiments. Statistical significance between unstimulated and stimulated cells was assessed using a paired t-test (**p < 0.01; ***p < 0.001; ns, not statistically significant)

We assessed β-arrestin 2 recruitment to the receptor using BRET^1^ assay. The assay measures the interaction between β-arrestin 2 fused to the Renilla reniformis luciferase (RLuc) energy donor and the receptor fused to fluorescent Venus energy acceptor (Fig. 3a). The recruitment of cytosolic β-arrestin 2 to the receptor at the plasma membrane in the presence of an agonist will promote a significant increase of the BRET signal compared to the basal state, reflecting the close proximity between the β-arrestin 2 energy donor and the receptor energy acceptor [22].

Interestingly, at basal state, we detected a significant BRET signal between P2Y_1_-R-Venus and β-arrestin 2-RLuc, indicating that P2Y_1_-R and β-arrestin 2 are already associated prior any agonist stimulation (Fig. 3b), thus demonstrating the constitutive activity of P2Y_1_-R on β-arrestin 2 pathway. Conversely, in similar conditions, P2Y_12_-R did not exhibit such a constitutive interaction with β-arrestin 2. As observed for P2Y_1_-R-dependent constitutive Gq protein activation (Additional File 2: Fig. S2a), the basal interaction between P2Y_1_-R—but not P2Y_12_-R—and β-arrestin 2 was also detected in the presence of high concentration of apyrase (Additional File 5: Fig. S5), thereby precluding any ADP-dependent β-arrestin 2 recruitment at P2Y_1_-R due to ADP release in the medium. To further confirm the specificity of P2Y_1_-R/β-arrestin 2 constitutive interaction, we performed BRET saturation curves in HEK293T cells co-expressing a fixed amount of the energy donor β-arrestin 2-RLuc and an increasing amount of the energy acceptor P2Y_1_-R-Venus. We detected a basal BRET signal between P2Y_1_-R-Venus and β-arrestin 2-RLuc that increased hyperbolically and saturated at high P2Y_1_-R-Venus concentration, thus demonstrating the specificity of the BRET signal (Fig. 3c). By contrast, a very weak BRET signal was detected when experiments were performed with P2Y_12_-R-Venus or soluble Venus as a negative control. This BRET signal is independent of the Venus expression level leading to linear curves, most likely reflecting bystander, nonspecific BRET signal (random collision) (Fig. 3c).

Surprisingly, in cells expressing P2Y_1_-R or P2Y_12_-R, ADP stimulation did not trigger β-arrestin 2 recruitment (Fig. 3d). By contrast and as expected, in AT1-R-expressing cells, Angiotensin II (AngII) promoted a time-dependent β-arrestin 2 recruitment (Fig. 3e). Importantly, MRS2179 significantly decreased the BRET signal between P2Y_1_-R-Venus and β-arrestin 2-RLuc, but not with P2Y_12_-R, reflecting a dissociation between P2Y_1_-R and β-arrestin 2 and thus demonstrating that MRS2179 behaved as a specific inverse agonist at P2Y_1_-R on the β-arrestin 2 recruitment (Fig. 3f). To further characterize the inverse agonist potency of MRS2179 at P2Y_1_-R, we performed a concentration-response curve on β-arrestin 2 recruitment and observed that much like on Gαq signaling (Fig. 1f), MRS2179 exhibited a low potency in the micromolar range (EC50 = 4.561 μM) (Fig. 3g).

Since GPCR constitutive activity is known to correlate with receptor expression level (Fig. 1d–e), we then monitored β-arrestin 2 recruitment in cells expressing decreasing amount of P2Y_1_-R. We found that decreasing receptor expression unveiled a significant agonist efficacy of ADP on β-arrestin 2 recruitment, with a concomitant loss of the inverse agonist efficacy of MRS2179 (Fig. 3h). Thus, decreasing receptor expression level—while keeping constant β-arrestin 2-RLuc probe expression (Additional File 6: Fig. S6a)—switched P2Y_1_-R from a constitutive active R* state targeted by MRS2179 to an inactive R and agonist-sensitive state, as reflected by the decrease of the basal BRET signal between P2Y_1_-R-Venus and β-arrestin 2-RLuc (Additional File 6: Fig. S6b). Accordingly, and in agreement with what we observed with ADP stimulation (Fig. 3h), the potent purinergic agonist 2-methylthioadenosine diphosphate (2MeSADP) only promoted β-arrestin 2 recruitment in cells expressing low levels of P2Y_1_-R (Additional File 6: Fig. S6c).

Altogether, these results strongly supported that at basal state, and at high expression level, P2Y_1_-R displayed a constitutive activation of β-arrestin 2 signaling and that MRS2179 behaved as an inverse agonist to counteract receptor-dependent constitutive β-arrestin 2 recruitment.

### P2Y_1_-R antagonists behaved as inverse agonists

**Fig. 4:**
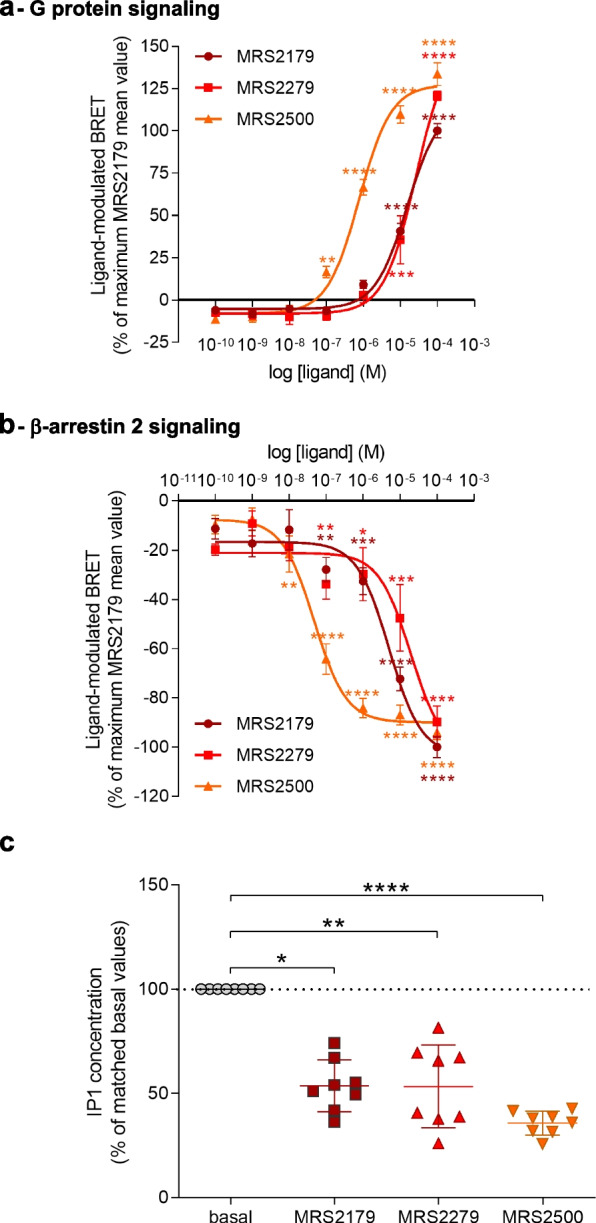
P2Y_1_-R antagonists behave as inverse agonists. **a** Dose-response curve was performed in HEK293T cells co-expressing Gαq-RLuc8, GFP2-Gγ2, Gβ1, and P2Y_1_-R after stimulation or not with increasing concentrations of MRS2179, MRS2279, or MRS2500 for 1 min. Results are expressed as the difference in the BRET signal measured in the presence and in the absence of ligand and are normalized to the mean value of maximal MRS2179 response. Data represent the mean ± s.e.m. of six independent experiments. Statistical significance between unstimulated and stimulated cells was assessed by one-way ANOVA followed by Sidak’s post-tests (**p < 0.01; ***p < 0.001; ****p < 0.0001). **b** Dose-response curve was performed in HEK293T cells co-expressing β-arrestin 2-RLuc and P2Y_1_-R-Venus after stimulation or not with increasing concentrations of MRS2179, MRS2279, or MRS2500 for 15 min. Results are expressed as the difference in the BRET signal measured in the presence and in the absence of ligand and are normalized to the mean value of maximal MRS2179 response. Data represent the mean ± s.e.m. of five independent experiments. Statistical significance between unstimulated and stimulated cells was assessed by one-way ANOVA followed by Sidak’s post-tests (*p < 0.05; **p < 0.01; ***p < 0.001; ****p < 0.0001). **c** Washed human platelets were incubated in the absence (basal) or in the presence of MRS2179 (10 μM), MRS2279 (10 μM), or MRS2500 (10 μM) for 2 h and IP1 accumulation was quantified. Data represent the mean ± s.d. of 8 healthy donors and are expressed as the percentage of matched basal values. The statistical comparison was assessed using Kruskal-Wallis test followed by Dunn’s post-tests (*p < 0.05; **p < 0.01; ****p < 0.0001)

We further deeply depicted the pharmacological signature of P2Y_1_-R by testing two other receptor antagonists. Thus, we explored the pharmacological properties of MRS2279 and MRS2500 and compared to MRS2179 by performing new concentration-response curve experiments on both Gαq protein activity and β-arrestin 2 recruitment in HEK293T cells. We observed that like MRS2179, the two P2Y_1_-R antagonists MRS2279 and MRS2500 behaved as inverse agonists on both P2Y_1_-R-dependent constitutive Gαq protein (Fig. 4a) and β-arrestin 2 (Fig. 4b) signaling.

In particular, we observed that although the three compounds displayed comparable maximal efficacy (Emax) on both constitutive Gαq protein activation and β-arrestin 2 recruitment, MRS2500 exhibited a greater potency than MRS2179 and MRS2279 (Table 1). Noteworthy, while MRS2179 had comparable potencies on both Gαq protein and β-arrestin 2 signaling, MRS2500 appeared to be more potent on β-arrestin 2 signaling than on Gαq protein signaling (Table 1), indicating that MRS2500 behaved as a biased inverse agonist compared to MRS2179.

**Table 1:** Maximal efficacies (Emax) and potencies (EC50) of MRS2179, MRS2279 and MRS2500 on P2Y_1_-R-dependent Gαq protein activation and β-arrestin 2 recruitment

**G protein signaling**			
	**Emax ± s.e.m.**	**EC50 (M)**	**pEC50 ± s.e.m.**
**MRS2179**	115.7 ± 5.057	1.525e−005	4.817 ± 0.0671
**MRS2279**	154.3 ± 13.86	2.638e−005	4.579 ± 0.1247
**MRS2500**	127.2 ± 3.892	7.716e−007	6.113 ± 0.07237
**β-Arrestin 2 signaling**			
	**Emax ± s.e.m**	**EC50 (M)**	**pEC50 ± s.e.m**
**MRS2179**	− 103.3 ± 6.549	5.055e−006	5.296 ± 0.153
**MRS2279**	− 102.8 ± 14.98	1.943e−005	4.712 ± 0.2838
**MRS2500**	− 89.95 ± 2.887	4.755e−008	7.323 ± 0.1193

Data were obtained from experiments shown in Fig. 4a, b and represent the Emax ± s.e.m, EC50 (M), and pEC50 ± s.e.m. on P2Y_1_-R-dependent Gαq protein and β-arrestin 2 constitutive signaling

Altogether, these results demonstrated the different pharmacological signatures of the three molecules at P2Y_1_-R constitutive activity.

Importantly, we also validated the physiological relevance of those results in human washed platelets and showed that as observed for MRS2179, MRS2279, and MRS2500 also exhibited inverse agonist efficacy on P2Y_1_-R-dependent constitutive Gαq signaling in human platelets with physiological expression levels of both endogenous P2Y_1_-R and G proteins (Fig. 4c).

## Discussion

In this study, we demonstrated for the first time the constitutive P2Y_1_-R signaling on PLC/IP3 pathway and the inverse agonist efficacy of MRS2179 in both HEK293T cells and mouse and human resting platelets. Interestingly, P2Y_12_-R, another ADP receptor playing a key role in platelet aggregation, also displayed constitutive signaling in human platelets [16, 17], emphasizing the importance of basal activation of ADP receptors in platelet physiology. As already suggested for constitutive P2Y_12_-R/Gi/o activation, we can speculate that at resting state, platelets already exhibit basal “low noise” P2Y_1_-R/Gq signaling that could not initiate *per se* platelet activation but that would ensure a rapid response to vascular injury.

**Fig. 5:**
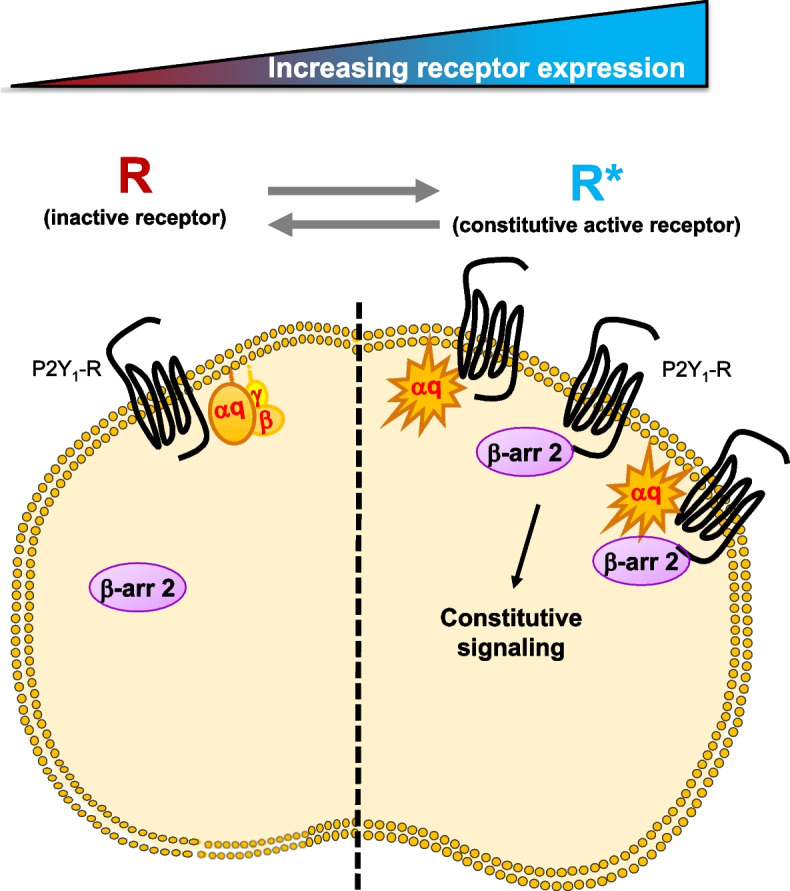
P2Y_1_-R constitutive activity depends on receptor expression level. Increasing P2Y_1_-R expression switched the receptor equilibrium from an inactive R conformation to an active R* conformation, thereby promoting a constitutive activation of Gq protein signaling as well as constitutive recruitment of β-arrestin 2, and precluding ADP-induced signaling. By contrast, decreasing P2Y_1_-R expression had an opposite effect, balancing the receptor equilibrium towards an inactive R state responding to agonist stimulation

Moreover, we showed that P2Y_1_-R—but not P2Y_12_-R—also exhibited constitutive interaction with β-arrestin 2. β-arrestins are key players of GPCR function and were primarily proposed to turn off GPCR signaling by triggering receptor desensitization and internalization [27]. Further studies then reported that β-arrestins also acted as signal transducers by scaffolding signaling complexes leading to the activation of signaling pathways, including MAPK signaling [28]. Therefore, constitutive basal P2Y_1_-R/β-arrestin 2 interaction could ultimately regulate receptor internalization and therefore affect receptor intracellular location and signaling capabilities. While the role of P2Y_1_-R/Gq signaling was well described in platelet aggregation and closely related to shape change through an increase in intracellular calcium triggered by Gq/PLC activation, the β-arrestin 2 signaling was contrastingly poorly documented. Recently, β-arrestins were described as negative regulators of GPCR signaling, acting like a brake on platelet aggregation [29]. In the literature, conflicting reports described either β-arrestin-dependent or β-arrestin-independent P2Y-R internalization and/or desensitization. Thus, Mundell and colleagues failed to observe β-arrestin 2 recruitment in P2Y_1_-R-expressing cells following ADP stimulation but showed an ADP-induced β-arrestin 2 translocation at P2Y_12_-R [30, 31]. In marked contrast, P2Y_1_-R was shown to promote ADP-induced β-arrestin 2 recruitment to the plasma membrane while P2Y_12_-R did not [32]. These discrepancies between different studies might be directly related to P2Y_1_-R expression level. Indeed, as already reported in the literature [23], GPCR constitutive activity is known to rise as a function of increasing receptor expression. Accordingly, we demonstrated here that increasing P2Y_1_-R expression triggered a constitutive association of the receptor with β-arrestin 2, precluding ADP-induced β-arrestin 2 recruitment while decreasing P2Y_1_-R expression had an opposite effect, balancing the receptor equilibrium towards an inactive population responding to agonist stimulation and promoting β-arrestin 2 recruitment (Figs. 3h and 5 and Additional File 6: Fig. S6c).

So far, whether the constitutive P2Y_1_-R activation on both G protein and β-arrestin 2 pathways relies solely on a unique receptor population or two separate ones still remains an open question. Thus, understanding the molecular basis of constitutively active P2Y_1_-R appears as a tremendous challenge to decipher the role of basal P2Y_1_-R signaling in platelet activation. One may envision that constitutive P2Y-R activity was associated with some receptor populations physically restricted to special membrane domains. GPCR organization at the cell surface relies on a dynamic equilibrium between monomers, dimers, and high-order oligomers [33]. Over the past years, a growing body of evidence reported that oligomerization could influence membrane expression, trafficking, and functional activity of GPCR, sometimes even generating novel pharmacological and signaling properties [34]. More recently, CXCR4 dimerization was closely linked to its basal activity [35]. A CXCR4 mutant displaying no basal activity was monomeric, supporting a positive correlation between CXCR4 basal signaling and dimeric organization. Interestingly, inverse agonists efficiently reduced CXCR4 basal activity but also abolished receptor dimerization [36].

In living cells, P2Y_1_-R and P2Y_12_-R exist as different oligomeric states and are capable to form not only homo-oligomers but also hetero-oligomers [37, 38]. Since previous studies demonstrated reciprocal cross-talk between P2Y_1_-R and P2Y_12_-R signaling [39], one can suggest that P2Y_1_-R/P2Y_12_-R dimers or oligomers could regulate basal constitutive activation of both receptors to fine-tune the purinergic signaling in platelets.

Furthermore, an increasing number of studies suggests that membrane components—i.e. lipids—surrounding GPCR may modulate receptor oligomeric states and thus possibly regulate basal receptor signaling [40]. P2Y_1_-R and P2Y_12_-R partitioned, at least in part, into cholesterol-enriched raft domains [37, 41]. Interestingly, in vivo clopidogrel treatment mostly converted P2Y_12_-R oligomers into dimers that partitioned outside the lipid rafts in freshly isolated platelets [37]. In contrast to cangrelor and ticagrelor, it is not known if clopidogrel behaves as an inverse agonist at P2Y_12_-R/Gi/o signaling, but it is tempting to speculate that ADP receptors inserted into raft domains undergo conformational constraints favoring oligomerization and constitutive activity. Inverse agonists including antiplatelet drugs could then inhibit basal receptor activation, by releasing GPCR out of these domains, possibly by disrupting oligomerization. In this context, inverse agonists might be an interesting field for future investigations to develop new therapeutic molecules able to modulate selectively the constitutive activity of P2Y receptors.

The physiological relevance of GPCR constitutive activity was unveiled in many biological processes as alterations of agonist-independent receptor signaling were associated with various diseases [42]. Accordingly, P2Y_12_-R displayed increased expression and constitutive activation in subjects with diabetes mellitus that exhibit platelet hyperactivity and high thrombotic risk [18]. By contrast, the bleeding syndrome-related R122C mutation of P2Y_12_-R correlated with a loss of constitutive receptor signaling [17]. Hence, monitoring P2Y_12_-R as well as P2Y_1_-R constitutive activation might be an early marker of platelet hyper-reactivity—usable in clinical practice—that could be directly associated with the bleeding or thrombotic risks. This should be particularly pertinent for the prediction/management of thrombosis in elderly patients as age-related diseases, including cardiovascular diseases, type 2 diabetes and Alzheimer’s disease, are associated with dysregulated platelet functions, platelet hyperactivity, enhanced aggregation, and/or increased risk of thrombotic events [43–45]. In this context, the reclassification of P2Y_1_-R antagonists into inverse agonists could have important pharmacological and therapeutic applications.

Apart from platelets, P2Y_1_-R also achieved many additional roles in other cell types, and particularly throughout the brain in neurons, astrocytes, and microglia. Interestingly, astrocyte hyperactivity, which is an important contributor to neuronal-glial network dysfunction in Alzheimer’s disease, was driven by enhanced P2Y_1_-R expression and activity [46]. Importantly, P2Y_1_-R inhibition with MRS2179 restored network homeostasis and protected from the decline of spatial learning and memory in an Alzheimer’s disease mouse model [47], thereby highlighting P2Y_1_-R as a novel target in the treatment of Alzheimer’s disease. Similarly, in epileptic models, brain P2Y_1_-R expression is increased [48] and correlated with an abnormal pattern of intracellular calcium oscillations. In this model, P2Y_1_-R antagonists normalize the duration of astroglial calcium oscillations and protect against seizure-induced cortical damages [48, 49]. Although those defects of intercellular calcium waves were primarily attributed to a release of P2Y_1_-R agonist both in Alzheimer’s disease and in epilepsy, we cannot exclude that such modifications of P2Y_1_-R expression levels can directly affect basal signaling of the receptor. Indeed, neuronal dysfunction and astrocyte hyperactivity might be directly linked to a detrimental increased P2Y_1_-R expression, and consequently enhanced basal P2Y_1_-R/Gq signaling, that would be responsible, at least in part and independently of agonist stimulation, for the increase in spontaneous astroglial calcium events. In this context, basal P2Y_1_-R constitutive activity could be an important feature to evaluate in the receptor pharmacological signature as it could unexpectedly contribute to disease pathogenesis.

## Conclusions

In recent years, the understanding of GPCR pharmacology and ligand efficacy opened up new avenues for GPCR drug discovery strategies. In particular, accumulating data have provided unequivocal evidences for the physiological relevance of the ligand-independent constitutive activation of GPCR and demonstrated the therapeutic value of modulating the constitutive activity by inverse agonists. In the case of the P2Y_12_ receptor, its constitutive activation started to gain interest since it was associated with high thrombotic risk when enhanced in diabetes patients or correlated with bleeding syndrome when abolished.

Here, we demonstrated that P2Y_1_-R also exhibits constitutive signaling in human platelets and that MRS2179, MRS2279 and MRS2500 behave as inverse agonists. Since this ligand-independent P2Y_1_-R constitutive activity is closely related to receptor expression level, monitoring P2Y_1_-R expression and constitutive activation could be a promising readout to evaluate the thrombotic risk in platelets and also in other cell types as P2Y_1_-R functions in a broad range of tissues. In the future, the development of selective inverse agonists of P2Y_1_-R might be a powerful strategy for antiplatelet therapy as well as for treatments for neurodegenerative disorders.

## Methods

### Materials

Human P2Y_1_-R (NCBI Reference Sequence: NM_002563.2) and P2Y_12_-R (NCBI Reference Sequence: NM_022788.3) were fused to double Myc epitope and rat AT1-R (NCBI Reference Sequence: NM_030985.4) to HA tag in N-terminus extracellular region. Alternatively, receptors were also fused to Venus tag in C-terminus intracellular region. Plasmids encoding Gαq-RLuc8, GFP2-Gγ2 and untagged Gβ1 were previously described [17, 21, 22]. ADP was purchased from Sigma-Aldrich/Merck (Darmstadt, Germany). MRS2179, MRS2279, MRS2500, and 2MeSADP were purchased from Tocris Bioscience (Bristol, UK) and luciferase substrates (coelenterazine 400a and h) from Interchim (Los Angeles, CA, USA). The Gq inhibitor (FR900359) was kindly supplied by Evi Kostenis (University of Bonn. Germany).

### Cell culture and transfection

Human embryonic kidney HEK293T/17 cells (ATCC) were maintained in DMEM AQmedia (Sigma-Aldrich/Merck, Darmstadt, Germany) supplemented with 10% fetal bovine serum (Life technologies) and 100 units/mL penicillin and 100 μg/mL streptomycin at 37 °C in a humidified atmosphere containing 5% CO_2_. Twenty-four hours after cell splitting, transient transfections were performed using polyethylenimine (PEI, Polysciences Inc.) according to the manufacturer’s instructions.

### Bioluminescence resonance energy transfer (BRET) measurements

G protein activation and β-arrestin 2 recruitment were performed as previously described [17, 21, 22]. Briefly, vectors encoding receptors, Gαq-RLuc8, GFP2-Gγ2, Gβ1, or β-arrestin 2 were transiently cotransfected into HEK293T/17 cells as indicated in the figure legends. Forty-eight hours after transfection, cells were washed and resuspended in PBS containing 0.1% (w/v) glucose at room temperature and then distributed (80 μg proteins/well) in a 96-well microplate (PerkinElmer). For G protein activation experiments (BRET^2^), cells were incubated in the absence (basal BRET signal) or in the presence of ligand for 1 minute. BRET signal between RLuc8 and GFP2 was measured after addition of the luciferase substrate coelenterazine 400a (5 μM). For β-arrestin 2 recruitment (BRET^1^), cells were stimulated with ligands for 5 or 15 min. BRET signal was recorded after incubation with coelenterazine h (5 μM) for 8 min. For kinetics studies, BRET signal between RLuc and Venus was recorded at 20-s intervals at basal state for the 2 first minutes and then for 5 min after ligand injection. For saturation curves (BRET^1^), the expression level of Venus- or RLuc-tagged protein was determined by direct measurement of total fluorescence and luminescence respectively. Total fluorescence was first measured with an excitation filter at 485 nm and an emission filter at 520 nm. Then, the same sample was incubated for 8 minutes with 5 μM coelenterazine h and the total luminescence was measured. BRET^2^ and BRET^1^ readings were collected using a modified Infinite F500 (Tecan Group Ltd). The BRET^2^ signal was calculated by the ratio of emission of GFP2 (510–540 nm) to RLuc8 (370–450 nm) and the BRET^1^ signal by the ratio of emission of Venus (520–570 nm) to RLuc (370–480 nm). Sometimes, results are expressed as the Net BRET computed by deducting the BRET background signal (obtained in the presence of the energy donor alone) from the BRET signal (acquired from cells expressing both the energy donor and acceptor).

### Quantification of cell surface receptors by ELISA

Cell surface receptor quantification was performed as previously described [17]. Briefly, HEK293T/17 cells were split into 24-well plates pre-coated with Poly-D-lysine, transiently transfected with a control empty vector (pcDNA3.1) or increasing amounts of vector encoding N-terminally Myc-tagged P2Y_1_-R or HA-tagged AT1-R. Forty-eight hours post-transfection, cells were fixed (1% paraformaldehyde), saturated (PBS–1% BSA) and incubated with the primary anti-Myc antibody (Clone 9E10. 1:500. Santa Cruz Biotechnology. Dallas, Texas, USA) or anti-HA (Clone 16B12. 1:2500. BioLegend. San Diego. California, USA) and then with HRP-labeled secondary antibody (Sigma-Aldrich/Merck. 1:1000. Darmstadt, Germany). After washing, cells were incubated for 15 min with HRP substrate: TMB (3,39,5,59-tetramethylbenzidine) (BD Biosciences). The reaction was stopped with HCl 1N, and the plates were read at 450 nm in a microplate reader (Infinite F500. Tecan Group Ltd. Männedorf, Switzerland).

### Animals

Two-month-old C57BL/6JRccHsd male mice (Envigo) were used for experiments and housed in the Anexplo (Toulouse) vivarium according to institutional guidelines. Ethical approval for animal experiments was obtained from the French Ministry of Research in agreement with European Union guidelines. Mice were housed in conventional cages under specific pathogen-free conditions in a constant temperature (20–22 °C) and humidity (50–60%) animal room with a 12/12 h light/dark cycle and free access to food and water.

### Washed murine platelets

Whole blood was drawn from the inferior vena cava of anesthetized mice (100 mg/kg ketamine, 10 mg/kg xylazine) into a syringe containing acid citrate dextrose (ACD) (1 volume anticoagulant/9 volumes blood). Platelet-rich plasma (PRP) was obtained by mixing blood with 1 volume of modified Hepes Tyrode’s buffer (137 mM NaCl, 2 mM KCl, 12 mM NaHCO_3_, 0.3 mM NaH_2_PO_4_, 1 mM MgCl_2_, 2 mM CaCl_2_, 5.5 mM glucose, 5 mM HEPES, and 0.35% (w/v) BSA, pH 6.7) followed by a centrifugation at 150 g for 2 min at 37 °C. Then, platelets were pelleted by centrifugation at 1000 g for 4 min, resuspended in modified Hepes Tyrode’s buffer (pH 7.4), adjusted to 3.10^8^ platelets/mL in the presence of 0.02 U/mL of apyrase and 10 μM indomethacin and rested for 45 minutes at 37°C before platelet stimulation. PGI_2_ (0.5 μM) was added before centrifugation steps to avoid platelet activation.

### Washed human platelets

Human platelets were prepared from adult healthy volunteers free of antiplatelet or anti-inflammatory medication since at least ten days. After informed consent, venous blood was collected, anticoagulated with 0.105 M citrate, and centrifuged (180 g, 10 min, room temperature) to obtain platelet-rich plasma (PRP). After two steps of centrifugation and suspension in Tyrode’s buffer (140 mM NaCl, 5 mM KCl, 5 mM KH_2_PO_4_, 1 mM MgSO_4_, 10 mM HEPES, 5 mM glucose and 0.35% (w/v) BSA, pH 6,7), washed platelets were suspended in the same buffer adjusted to pH 7.4 and containing 1 mM CaCl_2_. The final platelet suspension was adjusted to 3.10^8^ platelets/mL and rested for 45 min in the presence of 0.02 U/mL apyrase and 10 μM indomethacin at 37 °C prior experiments. PGI_2_ (0.5 μM) was added before centrifugation steps to avoid platelet activation.

### IP1 accumulation assay

Quantification of intracellular IP1 was performed using the HTRF (Homogeneous Time Resolved Fluorescence) IP1 competitive immunoassay (IP-One Tb kit. Cisbio. France) according to the manufacturer’s instructions. Briefly, 20,000 HEK293T/17 cells or 3.10^6^ platelets were distributed in a 384-well white microplate (Greiner) and incubated with the indicated molecules for 30 min or 2 h at 37 °C in the presence of 50 mM of LiCl to prevent IP1 degradation. After addition of d2-labeled IP1 (acceptor) and anti-IP1-Cryptate (donor) for 1 h, the specific FRET signals were calculated by the fluorescence ratio of the acceptor and donor emission signal (665/620 nm) collected using a modified Infinite F500 (Tecan Group Ltd). Conversion of the HTRF ratio of each sample into IP1 concentrations was performed on the basis of a standard curve to determine the linear dynamic range of the assay.

### Flow cytometry analysis

For platelet dense granule secretion, washed human platelets were stimulated with 50 μM TRAP (10 min, 37 °C) and stained with conjugated anti-CD63 FITC-conjugated antibody (BD Biosciences reference 557288. 1:5) for 15 min at room temperature. The platelets were then diluted into 1 mL PBS and samples were then kept in the dark until analysis by flow cytometry. The results are expressed as median fluorescence intensity (MFI).

### Data and statistical analysis

Statistical analysis was carried out using the GraphPad Prism 9.1 software (GraphPad Software Inc.). Statistical tests used are indicated in the figure legends. A *p* value < 0.05 was considered as significant.

## Supplementary Information

**Additional file 1: Fig. S1.** Relative expression of Gαq protein probe. a-b-c. Relative expression of Gαq-RLuc8 probe was assessed by luminescence measurement in Fig. 1b-c (a), in Fig. 1d (b) and in Fig. 1e (c). Data represent the mean ± s.e.m. of six (a) or five (b-c) independent experiments. (PPT 251 kb)

**Additional file 2: Fig. S2.** P2Y_1_-R constitutively activates Gq protein-dependent signaling in HEK293T cells in the presence of high apyrase concentration. a. Basal Gαq protein activation was evaluated by measuring basal BRET signal in HEK293T cells co-expressing Gαq-RLuc8, GFP2-Gγ2 and Gβ1 in the absence (pcDNA3.1) or in the presence of P2Y_1_-R after incubation or not with 0.2U/mL apyrase for 1, 5 or 10 minutes. Data represent the mean ± s.e.m. of four independent experiments and statistical significance between cells expressing P2Y_1_-R or not was assessed using one-way ANOVA followed by Sidak’s post-tests (*** *p* < 0.001). b. HEK293T cells expressing P2Y_1_-R, P2Y_12_-R or not (pcDNA3.1) were incubated in the presence of 0.2U/mL apyrase for 30 minutes or 2 hours and basal IP1 accumulation was quantified. Data represent the mean ± s.e.m of five independent experiments and are expressed as the percentage of the control mean (pcDNA3.1) at 30 minutes. The statistical comparison was assessed using one-way ANOVA followed by Sidak’s post-tests (*p < 0.05; **p < 0.01; ns, not statistically significant). (PPT 233 kb)

**Additional file 3: Fig. S3.** Relative receptor expression at the cell surface. a. HEK293T cells were transfected with increasing amounts of vectors encoding N-terminally Myc-tagged P2Y_1_-R (left panel) or HA-tagged AT1-R (right panel). Then, cell surface receptor expression was quantified. Data represent the mean ± s.e.m. of six independent experiments and results are expressed as the optical density (OD_450nm_) value after subtracting the background value obtained in control cells transfected with an empty vector (pcDNA3.1). Statistical significance was assessed by comparing the values obtained with receptor expression to the background value using one-way ANOVA followed by Dunnett’s post-tests (*p < 0.05; **p < 0.01; ***p < 0.001; ****p < 0.0001). b. HEK293T cells were transfected with N-terminally Myc-tagged P2Y_1_-R or P2Y_12_-R. Then, cell surface receptor expression was quantified. Data represent the mean ± s.e.m. of six independent experiments and results are expressed as the optical density (OD_450nm_) value after subtracting the background value obtained in control cells transfected with an empty vector (pcDNA3.1). Statistical significance between receptor expressions was assessed using an unpaired t-test (**p < 0.01). (PPT 225 kb)

**Additional file 4: Fig. S4.** P2Y_1_-R exhibits constitutive signaling in resting human platelets in the presence of high apyrase concentration. a. Washed human platelets were incubated in the presence of high concentration of apyrase (0.2U/mL) in the absence (basal) or in the presence of MRS2179 (10 μM) for 30 minutes or 2 hours and IP1 accumulation was quantified. Data represent the mean ± s.e.m. of 4 healthy donors and are expressed as the percentage of basal mean at 30 minutes. The statistical comparison between untreated (basal) and treated (MRS2179) platelets was assessed using one-way ANOVA followed by Sidak’s post-tests (*p < 0.05; ***p < 0.001). b. Secretion of platelet dense granules was assessed by flow cytometry using selective anti-CD63 antibody. Washed human platelets were analyzed either in resting conditions (basal) or following 10 minutes stimulation by TRAP (50 μM). Results are expressed as median fluorescence intensity (MFI) and data represent the mean ± s.e.m. of 4 healthy donors. Statistical analysis was performed using a paired t-test (**p < 0.01). (PPT 170 kb)

**Additional file 5: Fig. S5.** P2Y_1_-R constitutively associates with β-arrestin 2 in the presence of high apyrase concentration. Basal BRET signal was evaluated in HEK293T cells expressing β-arrestin 2-RLuc alone (pcDNA3.1) or together with P2Y_1_-R-Venus or P2Y_12_-R-Venus in the presence or not of 0.2U/mL apyrase. Data represent the mean ± s.e.m. of four independent experiments and statistical significance between cells expressing receptors or not was assessed using one-way ANOVA followed by Sidak’s post-tests (***p < 0.001; ns, not statistically significant). (PPT 133 kb)

**Additional file 6: Fig. S6.** Decreasing P2Y_1_-R cell surface expression unveils agonist-mediated β-arrestin 2 recruitment. a. Relative expression of β-arrestin 2-RLuc probe was assessed by luminescence measurement. Data represent the mean ± s.e.m. of five independent experiments. b. Basal BRET signal was evaluated in HEK293T cells expressing β-arrestin 2-RLuc and decreasing amounts of vectors encoding of P2Y_1_-R-Venus. Data represent the mean ± s.e.m. of five independent experiments. c. β-arrestin 2 recruitment was evaluated by monitoring BRET signal in HEK293T cells co-expressing β-arrestin 2-RLuc and decreasing amounts of vectors encoding P2Y_1_-R-Venus after stimulation or not with 2MeSADP (10 μM) for 15 minutes. Results are expressed as the difference in the BRET signal measured in the presence and in the absence of ligand. Data represent the mean ± s.e.m. of five independent experiments. Statistical significance between unstimulated and stimulated cells was assessed using a paired t-test (**p < 0.01; ns, not statistically significant).

**Additional file 7.** Source data for Figs 1-4.

**Additional file 8.** Source data for Figs S1-S6.

## Data Availability

All data generated and analyzed in this study are included in the supplementary information files of this published article. Source data for the main and supplementary figures is documented in Additional files 7-8.

## Abbreviations

2MeSADP: 2-Methylthioadenosine diphosphate
ADP: Adenosine diphosphate
BRET: Bioluminescence resonance energy transfer
cAMP: Cyclic adenosine monophosphate
GPCR: G protein-coupled receptor
HTRF: Homogeneous time resolved fluorescence
IP1: Inositol monophosphate
IP3: Inositol triphosphate
PLC: Phospholipase C
TRAP: Thrombin receptor activating peptide

## References

[1] Jin J, Kunapuli SP (1998). Coactivation of two different G protein-coupled receptors is essential for ADP-induced platelet aggregation. Proc Natl Acad Sci U S A.

[2] Jin J, Daniel JL, Kunapuli SP (1998). Molecular basis for ADP-induced platelet activation: II. The P2Y1 receptor mediates ADP-induced intracellular calcium mobilization and shape change in platelets. J Biol Chem.

[3] Hollopeter G, Jantzen HM, Vincent D, Li G, England L, Ramakrishnan V (2001). Identification of the platelet ADP receptor targeted by antithrombotic drugs. Nature.

[4] Franchi F, Angiolillo DJ (2015). Novel antiplatelet agents in acute coronary syndrome. Nat Rev Cardiol.

[5] Angiolillo DJ, Rollini F, Storey RF, Bhatt DL, James S, Schneider DJ (2017). International expert consensus on switching platelet P2Y12 receptor-inhibiting therapies. Circulation.

[6] Fabre JE, Nguyen M, Latour A, Keifer JA, Audoly LP, Coffman TM (1999). Decreased platelet aggregation, increased bleeding time and resistance to thromboembolism in P2Y1-deficient mice. Nat Med.

[7] Léon C, Hechler B, Freund M, Eckly A, Vial C, Ohlmann P (1999). Defective platelet aggregation and increased resistance to thrombosis in purinergic P2Y1 receptor-null mice. J Clin Investig.

[8] Lenain N, Freund M, Léon C, Cazenave JP, Gachet C (2003). Inhibition of localized thrombosis in P2Y1-deficient mice and rodents treated with MRS2179, a P2Y1 receptor antagonist. J Thrombosis Haemostasis.

[9] Hechler B, Nonne C, Eun JR, Cattaneo M, Cazenave JP, Lanza F (2006). MRS2500 [2-iodo-N6-methyl-(N)-methanocarba-2′- deoxyadenosine-3′,5′-bisphosphate], a potent, selective, and stable antagonist of the platelet P2y1 receptor with strong antithrombotic activity in mice. J Pharmacol Exp Ther.

[10] Chao H, Turdi H, Herpin TF, Roberge JY, Liu Y, Schnur DM (2013). Discovery of 2-(phenoxypyridine)-3-phenylureas as small molecule P2Y 1 antagonists. J Med Chem.

[11] Porto I, Giubilato S, de Maria GL, Biasucci LM, Crea F (2009). Platelet P2Y12 receptor inhibition by thienopyridines: Status and future. Expert Opin Investig Drugs.

[12] Serebruany VL, Sibbing D, Dinicolantonio JJ (2013). Dyspnea and reversibility of antiplatelet agents: ticagrelor, elinogrel, cangrelor, and beyond. Cardiology (Switzerland).

[13] Unverdorben M, Parodi G, Pistolesi M, Storey RF (2016). Dyspnea related to reversibly-binding P2Y12 inhibitors: a review of the pathophysiology, clinical presentation and diagnostics. Int J Cardiol.

[14] Gremmel T, Yanachkov IB, Yanachkova MI, Wright GE, Wider J, Undyala VVR (2016). Synergistic inhibition of both P2Y1 and P2Y12 adenosine diphosphate receptors as novel approach to rapidly attenuate platelet-mediated thrombosis. Arterioscler Thromb Vasc Biol.

[15] Koganov ES, Michelson AD, Yanachkov IB, Yanachkova MI, Wright GE, Przyklenk K (2018). GLS-409, an antagonist of both P2Y1 and P2Y12, potently inhibits canine coronary artery thrombosis and reversibly inhibits human platelet activation. Sci Rep.

[16] Aungraheeta R, Conibear A, Butler M, Kelly E, Nylander S, Mumford A (2016). Inverse agonism at the P2Y 12 receptor and ENT1 transporter blockade contribute to platelet inhibition by ticagrelor. Blood.

[17] Garcia C, Maurel-Ribes A, Nauze M, N’Guyen D, Martinez LO, Payrastre B (2019). Deciphering biased inverse agonism of cangrelor and ticagrelor at P2Y 12 receptor. Cell Mol Life Sci.

[18] Hu L, Chang L, Zhang Y, Zhai L, Zhang S, Qi Z (2017). Platelets express activated P2Y12 receptor in patients with diabetes mellitus. Circulation.

[19] Zhang Y, Ye J, Hu L, Zhang S, Zhang SH, Li Y (2012). Increased platelet activation and thrombosis in transgenic mice expressing constitutively active P2Y12. J Thrombosis Haemostasis.

[20] Pons V, Garcia C, Tidten-Luksch N, mac Sweeney A, Caroff E, Galés C (2022). Inverse agonist efficacy of selatogrel blunts constitutive P2Y12 receptor signaling by inducing the inactive receptor conformation. Biochem Pharmacol.

[21] Saulière A, Bellot M, Paris H, Denis C, Finana F, Hansen JT (2012). Deciphering biased-agonism complexity reveals a new active AT1 receptor entity. Nat Chem Biol.

[22] Galandrin S, Denis C, Boularan C, Marie J, M’Kadmi C, Pilette C (2016). Cardioprotective angiotensin-(1-7) peptide acts as a natural-biased ligand at the angiotensin II type 1 receptor. Hypertension.

[23] Milano CA, Allen LF, Rockman HA, Dolber PC, McMinn TR, Chien KR (1979). Enhanced myocardial function in transgenic mice overexpressing the β _2_ -adrenergic receptor. Science.

[24] Baurand A, Gachet C (2003). The P2Y1 receptor as a target for new antithrombotic drugs: a review of the P2Y1 antagonist MRS-2179. Cardiovasc Drug Rev.

[25] M’Kadmi C, Leyris JP, Onfroy L, Galés C, Sauliére A, Gagne D (2015). Agonism, antagonism, and inverse agonism bias at the ghrelin receptor signaling. J Biol Chem.

[26] Schrage R, Schmitz AL, Gaffal E, Annala S, Kehraus S, Wenzel D (2015). The experimental power of FR900359 to study Gq-regulated biological processes. Nat Commun.

[27] Kang DS, Tian X, Benovic JL (2014). Role of β-arrestins and arrestin domain-containing proteins in G protein-coupled receptor trafficking. Curr Opin Cell Biol.

[28] DeWire SM, Ahn S, Lefkowitz RJ, Shenoy SK (2007). β-Arrestins and cell signaling. Annual Rev Physiol.

[29] Hutchinson JL, Zhao X, Hill R, Mundell SJ (2020). Arrestin-3 differentially regulates platelet GPCR subsets. Platelets.

[30] Mundell SJ, Luo J, Benovic JL, Conley PB, Poole AW (2006). Distinct clathrin-coated pits sort different G protein-coupled receptor cargo. Traffic.

[31] Nisar S, Daly ME, Federici AB, Artoni A, Mumford AD, Watson SP (2011). An intact PDZ motif is essential for correct P2Y 12 purinoceptor traffic in human platelets. Blood.

[32] Hoffmann C, Ziegler N, Reiner S, Krasel C, Lohse MJ (2008). Agonist-selective, receptor-specific interaction of human P2Y receptors with β-arrestin-1 and -2. J Biol Chem.

[33] Dague E, Pons V, Roland A, Azaïs J-M, Arcucci S, Lachaize V, et al. Atomic force microscopy-single-molecule force spectroscopy unveils GPCR cell surface architecture. Commun Biol. 2022. 10.1038/s42003-022-03162-w. 10.1038/s42003-022-03162-w PMC8913689 35273337

[34] Milligan G, Ward RJ, Marsango S (2019). GPCR homo-oligomerization. Curr Opin Cell Biol.

[35] Perpiñá-Viciano C, Işbilir A, Zarca A, Caspar B, Kilpatrick LE, Hill SJ (2020). Kinetic analysis of the early signaling steps of the human chemokine receptor CXCR4. Mol Pharmacol.

[36] Isbilir A, Möller J, Arimont M, Bobkov V, Perpiñá-Viciano C, Hoffmann C (2020). Advanced fluorescence microscopy reveals disruption of dynamic CXCR4 dimerization by subpocket-specific inverse agonists. Proc Natl Acad Sci U S A.

[37] Savi P, Zachayus JL, Delesque-Touchard N, Labouret C, Hervé C, Uzabiaga MF (2006). The active metabolite of Clopidogrel disrupts P2Y12 receptor oligomers and partitions them out of lipid rafts. Proc Natl Acad Sci U S A.

[38] Choi RCY, Simon J, Tsim KWK, Barnard EA (2008). Constitutive and agonist-induced dimerizations of the P2Y1 receptor: Relationship to internalization and scaffolding. J Biol Chem.

[39] Hardy AR, Jones ML, Mundell SJ, Poole AW (2004). Reciprocal cross-talk between P2Y1 and P2Y12 receptors at the level of calcium signaling in human platelets. Blood.

[40] Gahbauer S, Böckmann RA (2016). Membrane-mediated oligomerization of G protein coupled receptors and its implications for GPCR function. Front Physiol.

[41] Norambuena A, Poblete MI, Donoso MV, Espinoza CS, González A, Huidobro-Toro JP (2008). P2Y1 receptor activation elicits its partition out of membrane rafts and its rapid internalization from human blood vessels: Implications for receptor signaling. Mol Pharmacol.

[42] Tao YX (2008). Constitutive activation of G protein-coupled receptors and diseases: Insights into mechanisms of activation and therapeutics. Pharmacol Ther.

[43] Stellos K, Panagiota V, Kögel A, Leyhe T, Gawaz M, Laske C (2010). Predictive value of platelet activation for the rate of cognitive decline in Alzheimers disease patients. Journal of Cerebral Blood Flow and Metabolism..

[44] Kim JH, Bae HY, Kim SY (2013). Clinical marker of platelet hyperreactivity in diabetes mellitus. Diabet Metab J.

[45] Jones CI (2016). Platelet function and ageing. Mammalian Genome.

[46] Delekate A, Füchtemeier M, Schumacher T, Ulbrich C, Foddis M, Petzold GC (2014). Metabotropic P2Y1 receptor signalling mediates astrocytic hyperactivity in vivo in an Alzheimer’s disease mouse model. Nat Commun.

[47] Reichenbach N, Delekate A, Breithausen B, Keppler K, Poll S, Schulte T (2018). P2Y1 receptor blockade normalizes network dysfunction and cognition in an Alzheimer’s disease model. J Exp Med.

[48] Alves M, Smith J, Engel T (2020). Differential expression of the metabotropic P2Y receptor family in the cortex following status epilepticus and neuroprotection via P2Y1 antagonism in mice. Front Pharmacol.

[49] Wellmann M, Álvarez-Ferradas C, Maturana CJ, Sáez JC, Bonansco C (2018). Astroglial Ca 2+ -dependent hyperexcitability requires p2y 1 purinergic receptors and pannexin-1 channel activation in a chronic model of epilepsy. Front Cell Neurosci.

