# Supplementary figures and images for: Platelet P2Y1 receptor exhibits constitutive G protein signaling and β-arrestin 2 recruitment
Source: BMC Biol. 2023 Feb 1;21:14. doi: 10.1186/s12915-023-01528-y (PMC9890698; doi:10.1186/s12915-023-01528-y)

## Slide 1
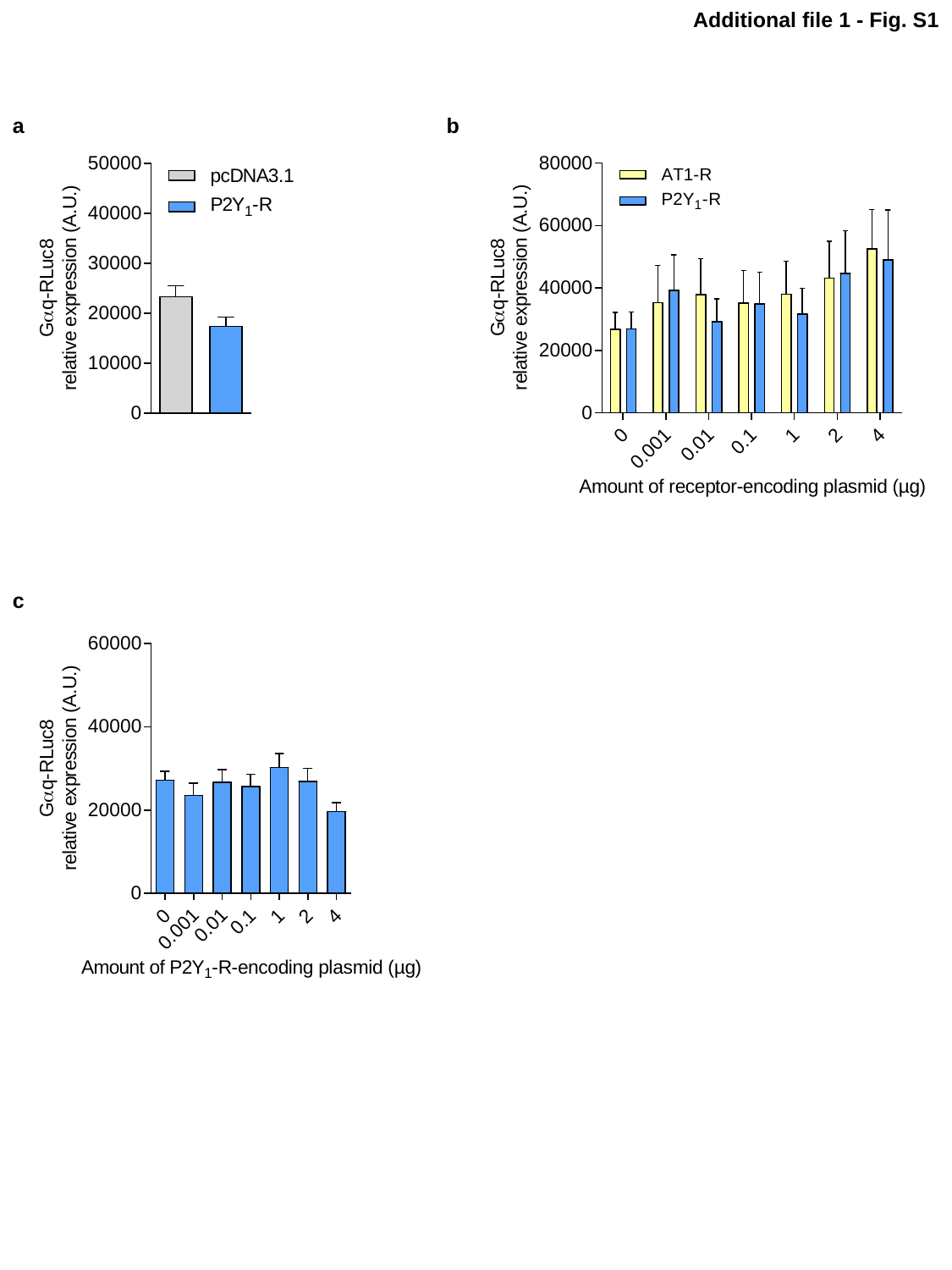

Additional file 1 - Fig. S1
a
b
c

Supplement: Supplementary file 1 — Additional file 1: Fig. S1. Relative expression of Gαq protein probe. a-b-c. Relative expression of Gαq-RLuc8 probe was assessed by luminescence measurement in Fig. 1b-c (a), in Fig. 1d (b) and in Fig. 1e (c). Data represent the mean ± s.e.m. of six (a) or five (b-c) independent experiments. (PPT 251 kb) [file 12915_2023_1528_MOESM1_ESM.ppt]

## Slide 1
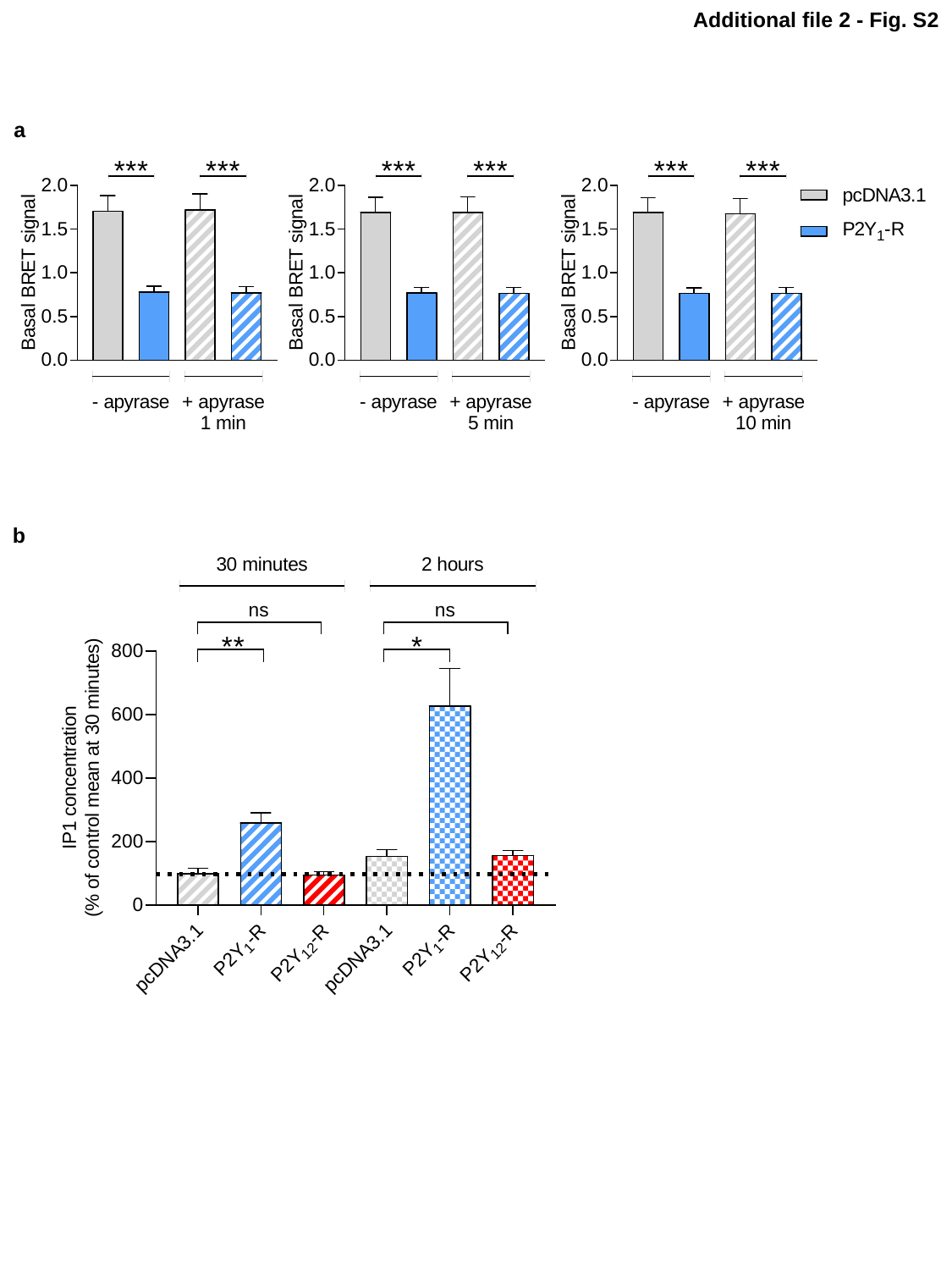

Additional file 2 - Fig. S2
a
b

Supplement: Supplementary file 2 — Additional file 2: Fig. S2. P2Y1-R constitutively activates Gq protein-dependent signaling in HEK293T cells in the presence of high apyrase concentration. a. Basal Gαq protein activation was evaluated by measuring basal BRET signal in HEK293T cells co-expressing Gαq-RLuc8, GFP2-Gγ2 and Gβ1 in the absence (pcDNA3.1) or in the presence of P2Y1-R after incubation or not with 0.2U/mL apyrase for 1, 5 or 10 minutes. Data represent the mean ± s.e.m. of four independent experiments and statistical significance between cells expressing P2Y1-R or not was assessed using one-way ANOVA followed by Sidak’s post-tests (*** p < 0.001). b. HEK293T cells expressing P2Y1-R, P2Y12-R or not (pcDNA3.1) were incubated in the presence of 0.2U/mL apyrase for 30 minutes or 2 hours and basal IP1 accumulation was quantified. Data represent the mean ± s.e.m of five independent experiments and are expressed as the percentage of the control mean (pcDNA3.1) at 30 minutes. The statistical comparison was assessed using one-way ANOVA followed by Sidak’s post-tests (*p < 0.05; **p < 0.01; ns, not statistically significant). (PPT 233 kb) [file 12915_2023_1528_MOESM2_ESM.ppt]

## Slide 1
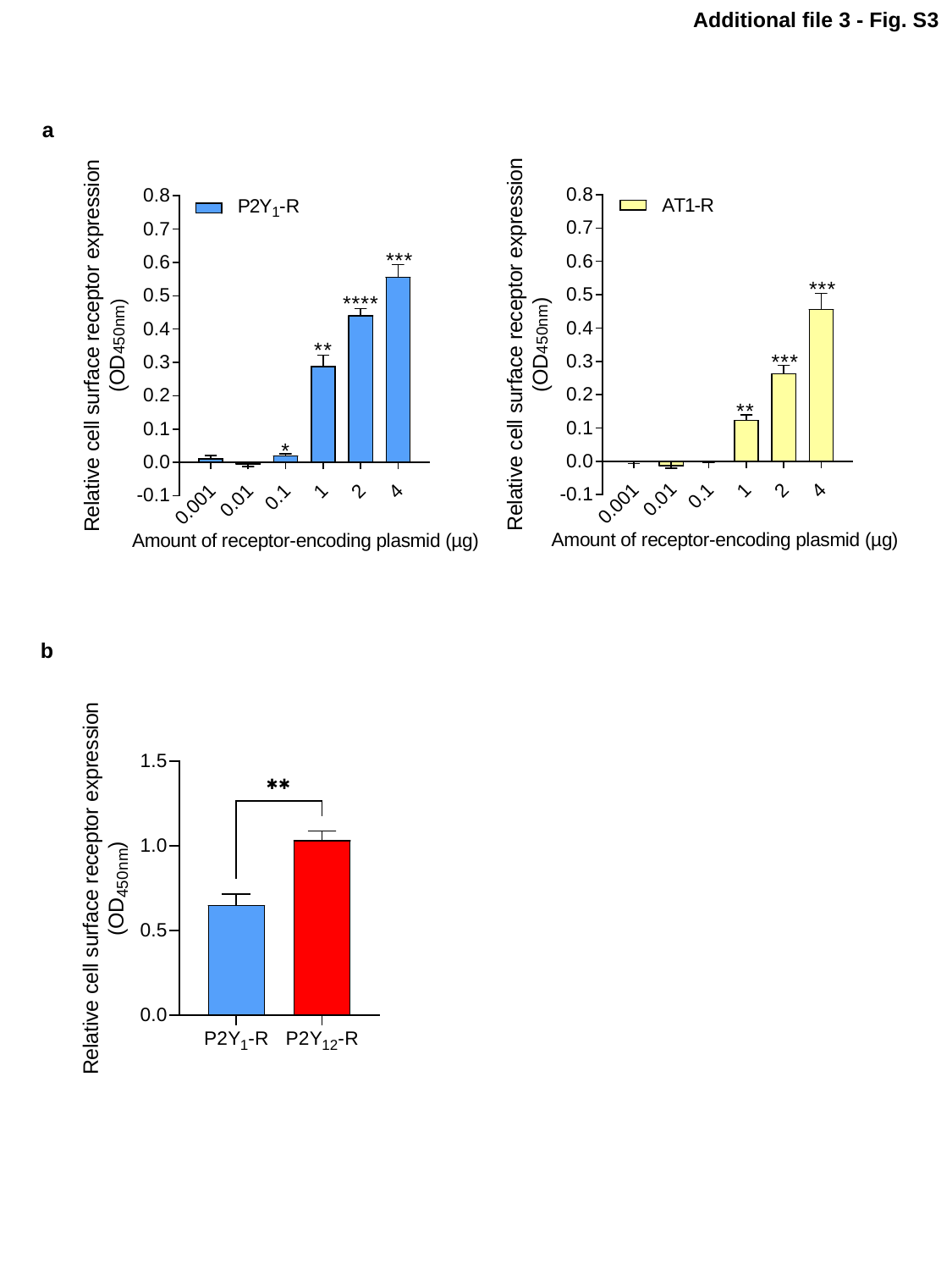

Additional file 3 - Fig. S3
a
b

Supplement: Supplementary file 3 — Additional file 3: Fig. S3. Relative receptor expression at the cell surface. a. HEK293T cells were transfected with increasing amounts of vectors encoding N-terminally Myc-tagged P2Y1-R (left panel) or HA-tagged AT1-R (right panel). Then, cell surface receptor expression was quantified. Data represent the mean ± s.e.m. of six independent experiments and results are expressed as the optical density (OD450nm) value after subtracting the background value obtained in control cells transfected with an empty vector (pcDNA3.1). Statistical significance was assessed by comparing the values obtained with receptor expression to the background value using one-way ANOVA followed by Dunnett’s post-tests (*p < 0.05; **p < 0.01; ***p < 0.001; ****p < 0.0001). b. HEK293T cells were transfected with N-terminally Myc-tagged P2Y1-R or P2Y12-R. Then, cell surface receptor expression was quantified. Data represent the mean ± s.e.m. of six independent experiments and results are expressed as the optical density (OD450nm) value after subtracting the background value obtained in control cells transfected with an empty vector (pcDNA3.1). Statistical significance between receptor expressions was assessed using an unpaired t-test (**p < 0.01). (PPT 225 kb) [file 12915_2023_1528_MOESM3_ESM.ppt]

## Slide 1
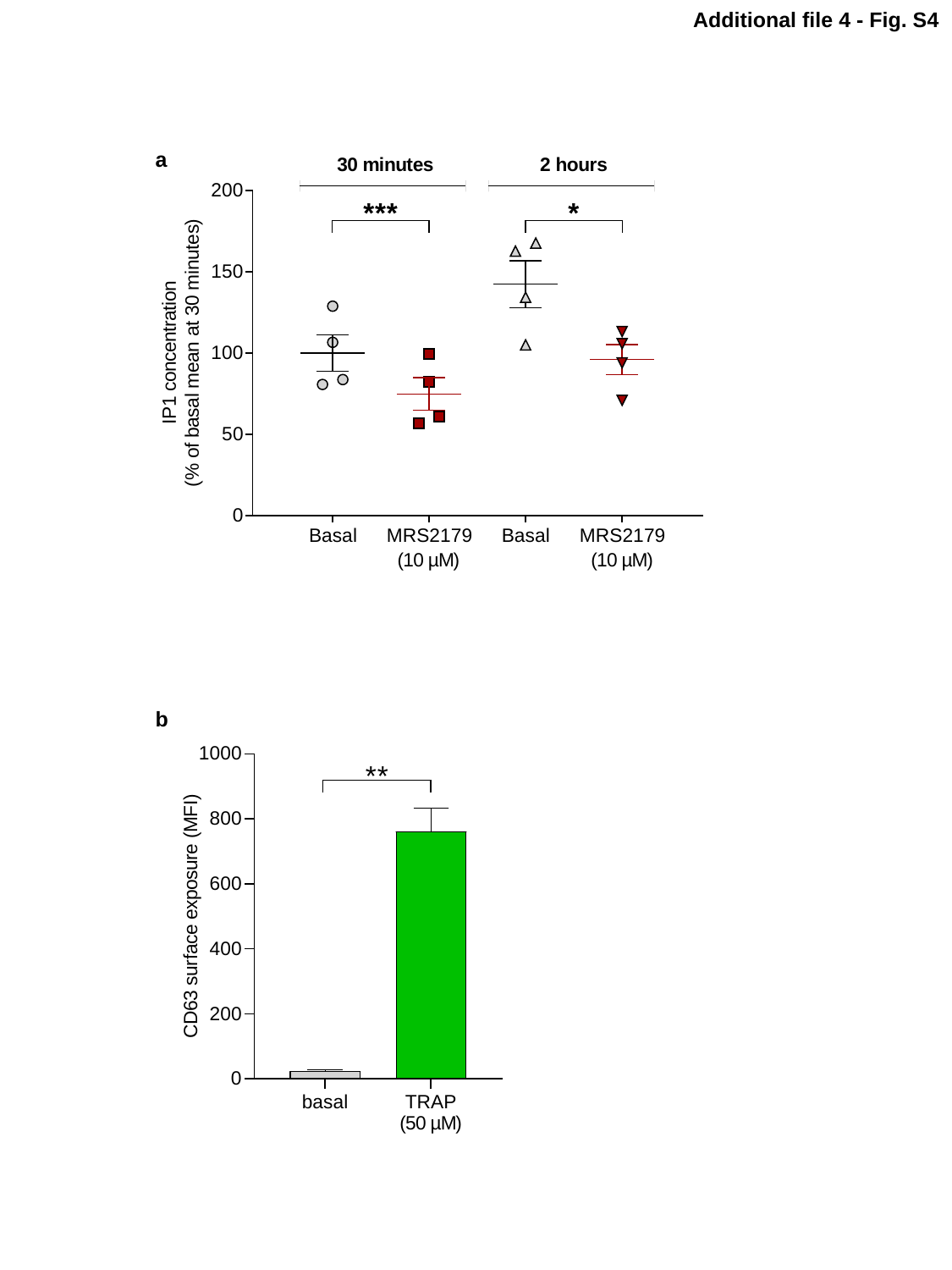

Additional file 4 - Fig. S4
a
b

Supplement: Supplementary file 4 — Additional file 4: Fig. S4. P2Y1-R exhibits constitutive signaling in resting human platelets in the presence of high apyrase concentration. a. Washed human platelets were incubated in the presence of high concentration of apyrase (0.2U/mL) in the absence (basal) or in the presence of MRS2179 (10 μM) for 30 minutes or 2 hours and IP1 accumulation was quantified. Data represent the mean ± s.e.m. of 4 healthy donors and are expressed as the percentage of basal mean at 30 minutes. The statistical comparison between untreated (basal) and treated (MRS2179) platelets was assessed using one-way ANOVA followed by Sidak’s post-tests (*p < 0.05; ***p < 0.001). b. Secretion of platelet dense granules was assessed by flow cytometry using selective anti-CD63 antibody. Washed human platelets were analyzed either in resting conditions (basal) or following 10 minutes stimulation by TRAP (50 μM). Results are expressed as median fluorescence intensity (MFI) and data represent the mean ± s.e.m. of 4 healthy donors. Statistical analysis was performed using a paired t-test (**p < 0.01). (PPT 170 kb) [file 12915_2023_1528_MOESM4_ESM.ppt]

## Slide 1
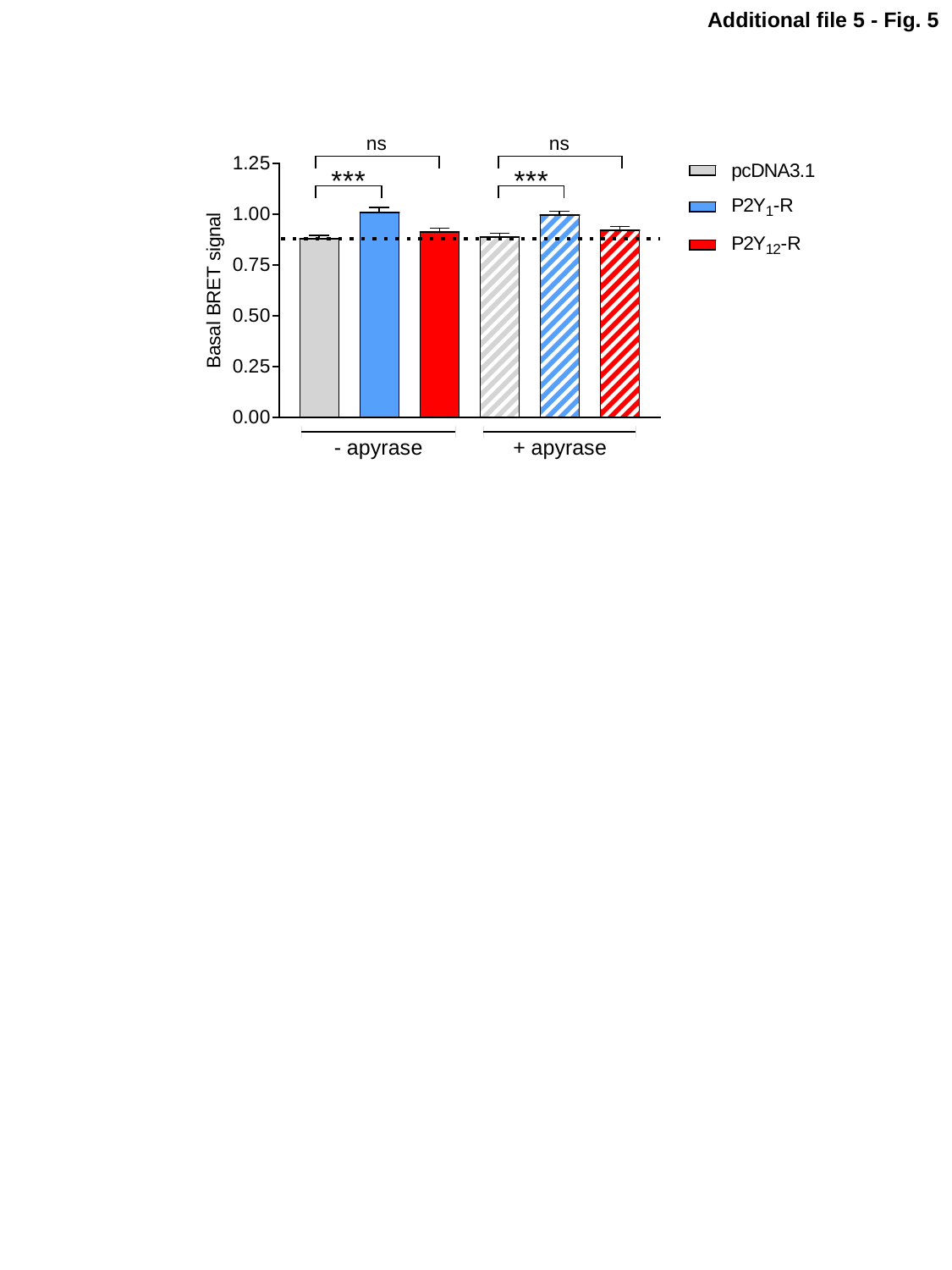

Additional file 5 - Fig. 5

Supplement: Supplementary file 5 — Additional file 5: Fig. S5. P2Y1-R constitutively associates with β-arrestin 2 in the presence of high apyrase concentration. Basal BRET signal was evaluated in HEK293T cells expressing β-arrestin 2-RLuc alone (pcDNA3.1) or together with P2Y1-R-Venus or P2Y12-R-Venus in the presence or not of 0.2U/mL apyrase. Data represent the mean ± s.e.m. of four independent experiments and statistical significance between cells expressing receptors or not was assessed using one-way ANOVA followed by Sidak’s post-tests (***p < 0.001; ns, not statistically significant). (PPT 133 kb) [file 12915_2023_1528_MOESM5_ESM.ppt]

## Slide 1
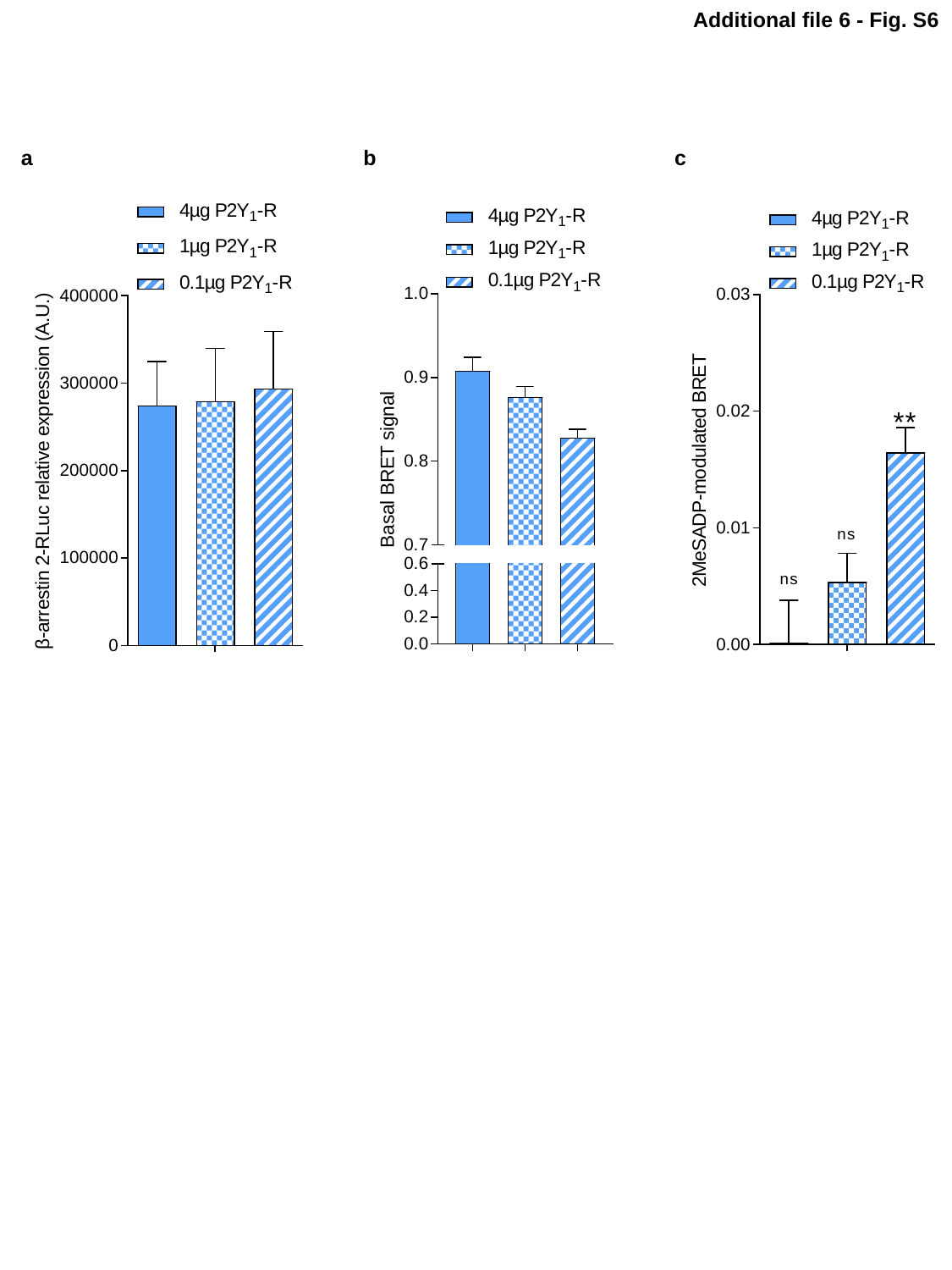

Additional file 6 - Fig. S6
a
b
c

Supplement: Supplementary file 6 — Additional file 6: Fig. S6. Decreasing P2Y1-R cell surface expression unveils agonist-mediated β-arrestin 2 recruitment. a. Relative expression of β-arrestin 2-RLuc probe was assessed by luminescence measurement. Data represent the mean ± s.e.m. of five independent experiments. b. Basal BRET signal was evaluated in HEK293T cells expressing β-arrestin 2-RLuc and decreasing amounts of vectors encoding of P2Y1-R-Venus. Data represent the mean ± s.e.m. of five independent experiments. c. β-arrestin 2 recruitment was evaluated by monitoring BRET signal in HEK293T cells co-expressing β-arrestin 2-RLuc and decreasing amounts of vectors encoding P2Y1-R-Venus after stimulation or not with 2MeSADP (10 μM) for 15 minutes. Results are expressed as the difference in the BRET signal measured in the presence and in the absence of ligand. Data represent the mean ± s.e.m. of five independent experiments. Statistical significance between unstimulated and stimulated cells was assessed using a paired t-test (**p < 0.01; ns, not statistically significant). [file 12915_2023_1528_MOESM6_ESM.ppt]
